# The Influence of Habitat on Viral Diversity in Neotropical Rodent Hosts

**DOI:** 10.3390/v13091690

**Published:** 2021-08-26

**Authors:** Sourakhata Tirera, Benoit de Thoisy, Damien Donato, Christiane Bouchier, Vincent Lacoste, Alain Franc, Anne Lavergne

**Affiliations:** 1 Laboratoire des Interactions Virus-Hôtes, Institut Pasteur de la Guyane, BP 6010, 97306 Cayenne, France; stirera@pasteur-cayenne.fr (S.T.); bdethoisy@pasteur-cayenne.fr (B.d.T.); ddonato@pasteur-cayenne.fr (D.D.); vincent.lacoste@pasteur.fr (V.L.); 2 Institut Pasteur, 25-28 Rue du Docteur Roux, CEDEX 15, 75724 Paris, France; bouchier@pasteur.fr; 3 Département de Virologie, Institut Pasteur, 75015 Paris, France; 4 Arbovirus & Emerging Viral Diseases Laboratory, Institut Pasteur du Laos, Vientiane 3560, Laos; 5 UMR BIOGECO, INRAE, University Bordeaux, 33612 Cestas, France; alain.franc@inra.fr; 6 Pleiade, EPC INRIA-INRAE-CNRS, University Bordeaux, 33405 Talence, France

**Keywords:** virome, alpha diversity, rodents, Amazonia, viral ecology, viral phylogenies

## Abstract

Rodents are important reservoirs of numerous viruses, some of which have significant impacts on public health. Ecosystem disturbances and decreased host species richness have been associated with the emergence of zoonotic diseases. In this study, we aimed at (a) characterizing the viral diversity in seven neotropical rodent species living in four types of habitats and (b) exploring how the extent of environmental disturbance influences this diversity. Through a metagenomic approach, we identified 77,767 viral sequences from spleen, kidney, and serum samples. These viral sequences were attributed to 27 viral families known to infect vertebrates, invertebrates, plants, and amoeba. Viral diversities were greater in pristine habitats compared with disturbed ones, and lowest in peri-urban areas. High viral richness was observed in savannah areas. Differences in these diversities were explained by rare viruses that were generally more frequent in pristine forest and savannah habitats. Moreover, changes in the ecology and behavior of rodent hosts, in a given habitat, such as modifications to the diet in disturbed vs. pristine forests, are major determinants of viral composition. Lastly, the phylogenetic relationships of four vertebrate-related viral families (*Polyomaviridae*, *Flaviviridae*, *Togaviridae*, and *Phenuiviridae*) highlighted the wide diversity of these viral families, and in some cases, a potential risk of transmission to humans. All these findings provide significant insights into the diversity of rodent viruses in Amazonia, and emphasize that habitats and the host’s dietary ecology may drive viral diversity. Linking viral richness and abundance to the ecology of their hosts and their responses to habitat disturbance could be the starting point for a better understanding of viral emergence and for future management of ecosystems.

## 1. Introduction

Viruses have conquered all living systems, infecting other microbes (bacteria, fungi, and parasites) and more complex organisms, such as plants, invertebrates, and vertebrates. Most viruses have small genomes with high mutation rates [1], giving them the ability to evolve and adapt quickly to new environments and potentially the ability to infect new hosts.

The development of metagenomic approaches applied to viruses (viromics) [2,3] has improved our knowledge of the extent of viral diversity and of the host spectra of several viral families. This is, for instance, the case for hepaciviruses, with both the descriptions of novel viral species in mammals and the evidence of infection in non-mammal species [4,5]. Viromic studies have also led to the discovery of new viral genotypes, helping us understand their evolutionary history [6] and providing new insights into the roles of viruses in ecosystems [7,8,9]. Despite these advances, a large number of viral species remain unknown and many biomes continue to be under- or unexplored [10]. In the context of natural habitat disturbances, the disruptions of population dynamics and ecology that favor contacts between species, cross-species transmissions, spill-over, amplification, spread of viruses, and increased contact with wild fauna may lead, under certain circumstances, to the emergence of infectious diseases (EIDs) in human populations [11,12,13]. It is now well established that more than 70% of EIDs originated from animals and mainly from wildlife [14]. Recent examples of virus spill-overs, such as the 2002 severe acute respiratory syndrome (SARS) in China and the 2012 Middle East respiratory syndrome (MERS) in Saudi Arabia, or even the 2014 Ebola epidemic in West Africa, have had severe public health and economic consequences [15,16]. The impact of the COVID-19 pandemic, though yet to be assessed, appears to be far more damaging [17]. Hence, both the identification of potentially zoonotic viruses, and the understanding of their transmission mechanisms and their ecological contexts, have gained attention in the last few decades [18], as illustrated by the PREDICT global surveillance program [19] and the Global Virome Project [20,21]. Despite limited knowledge of the processes of zoonotic viral emergence, several groups of species, such as primates, birds, bats, and rodents, are described as major reservoirs due to their ability to host wide ranges of viruses [22,23].

*Rodentia* is composed of approximately 2277 species [24,25] and is the most speciose mammalian order. They occupy most of the earth’s ecosystems, from highly anthropized to pristine natural habitats. The richness of rodent species, along with certain traits such as their fast-paced lives, population dynamics, opportunism, and synanthropism makes them efficient amplifiers, spreaders, and transmitters of viruses [23,26,27,28]. This is illustrated by the 31 mammalian viral families reported from the DRodVir online database, as of 21 July 2021 [29,30]. Rodents also play a key role in viral emergence phenomena, with many species being reservoirs and carriers of viral pathogens, for example, hantaviruses or mammarenaviruses, some of which are associated with human diseases [31,32,33,34,35].

Amazonia is known for its high biodiversity of mammals, plants, invertebrates, and microbes [36,37,38], comprising a large set of potential hosts for viruses. This implies that high viral diversity is expected from the region and thus a high number of potential zoonotic viruses [39,40]. Despite this context, Amazonia remains one of the least explored areas in terms of viral diversity, and to date, the few studies investigating the viral diversity of the main reservoir species have only been conducted with bats [8,41,42]. In French Guiana, 36 rodent species are present, and live in different habitats (various types of forest, savannah, agricultural areas, peri-urban zones, and urban zones). Among them, several species have been described to host viruses relevant to human health [33]. As an example, hantaviruses, belonging to the *Bunyavirales* order, are widespread worldwide and in South America cause a cardiopulmonary syndrome with a high mortality rate. In French Guiana, six human cases of cardiopulmonary syndrome have been identified to date, four of which were fatal [43,44,45]. Screening rodents captured in more likely areas of contamination made it possible to identify two species (*Zygodontomys brevicauda* and *Oligoryzomys delicatus*) as potential reservoirs of this virus [32]. To date, no human cases of mammarenavirus infection have been registered in French Guiana, although two members of this viral family (*Arenaviridae*), which in severe cases can cause hemorrhagic fevers and meningitis, were recently identified in rodents [46,47].

We first explored the viral diversity of seven Neotropical rodent species from two families: Echimyidae (*Proechimys cuvieri* and *P. guyannensis*) and Cricetidae (*Zygodontomys brevicauda*, *Oecomys bicolor*, *O. auyantepui*, *Hylaeamys yunganus*, and *Hylaeamys megacephalus*) to determine how viral diversity is distributed across species and habitats. Second, we focused on the phylogenetic relationships of viral sequences related to four viral families known to infect vertebrates including arthropod-borne viruses (*Polyomaviridae, Flaviviridae, Togaviridae*, and *Phenuiviridae*). These results allowed us to identify novel vertebrate-related viral sequences and shed new light on the role of habitats in shaping viral diversity in Amazonian rodent species.

## 2. Materials and Methods

### 2.1. Ethical and Legal Statements

All animals were captured, handled, and sampled following ASM guidelines under the supervision of researchers granted the French animal experimentation level 1 diploma [48]. The use of genetic resources was approved by the French Ministry of the Environment under reference number ABSCH-IRCC-FR-252439-1, 9 June 2020, in compliance with the Access and Benefit Sharing procedure implemented by the *Loi pour la Reconquête de la Biodiversité*.

### 2.2. Capturing

Within the framework of a long-term project dedicated to assessing the role of wildlife in the emergence of different viruses in French Guiana, rodents were sampled across the region from a total of 12 sites during the 2001–2014 period. Four environments were considered: disturbed and pristine forests, savannahs, and peri-urban habitats, according to the 2015 land use classification [49]. This classification was based on the Europe CORINE (coordinated information on the environment) Land Cover methodology [50] but was adapted to the country, since forms of urbanization and natural habitat categories differ greatly between continental Europe and French Guiana.

According to the sites, 20–30 trap-lines were organized in successive stations spaced 20 m apart with four traps at each station: two Sherman traps (Sherman Trap Co., Tallahassee, FL, USA) and two BTTm traps (BTTm, Besançon Trap Service mécanique, Besançon, France). The traps were baited with apples and checked every morning. The mean sampling effort per site was 1016 ± 420 traps/night, for a total effort of 28,470 traps/night and more than 500 rodent captures (belonging to 19 species) [33]. Rodents were caught alive, brought back to the laboratory facilities, anesthetized, and sampled for blood and/or euthanized to preserve their organs (kidney and spleen). When necessary, sacrifice was done chemically with barbituric acid after anesthesia, one of the recommended methods for wild-caught rodents, according to the AVMA Guidelines for the Euthanasia of Animals, 2020 Edition. The animals were identified using external morphological features and confirmed molecularly using the cytochrome oxydase I sequence [51,52]. Seven species were selected for the virome analysis according to their presence in distinct habitats: *P. cuvieri*, *P. guyannensis*, *Z. brevicauda*, *O. bicolor*, *O. auyantepui*, *H. yunganus*, and *H. megacephalus* (Table 1).

### 2.3. Sample Processing

Prior to processing, samples from the same species, the same organs, and the same environment were pooled (e.g., the sample k_Pguy_PF is a pool of kidneys from *P. guyannensis* collected in pristine forest), resulting in 36 different pools, i.e., 36 different viromes. Pools, at the organ level, included 2–31 individuals according to sample availability. Overall, 442 organs and sera from 187 individuals were included in this study (Table 1).

For serum samples, 50 µL from each collecting tube was used to constitute the pools. For kidney and spleen samples, 100 mg of organs was crushed in 400 µL of DMEM, and 200 µL of suspension from each collecting tube was used to constitute the pools. All pools were processed as previously described [41]: Pools were cleared of debris by low-speed centrifugation (5 min, 10,000× *g*, 4 °C). Eukaryotic and prokaryotic cell-sized particles were removed from supernatants through three successive filtrations (0.8, 0.45, and 0.22 μm), using cellulose acetate membrane filters (Nalgene). The filtrates were cleared of persistent high-density particles with low-speed centrifugation (15 min, 10,000× *g*, 4 °C); then, viral particles were pelleted with a 1-h ultracentrifugation step (100,000× *g*, 4 °C). All viral pellets were resuspended in 40 µL of nuclease-free water.

#### 2.3.1. Nuclease Treatment and Viral Nucleic Acid Extraction

All resuspended viral pellets were treated with a mixture of DNases (Turbo DNase from Ambion and Benzonase from Novagen) and RNase One (Promega) to digest non-enveloped nucleic acids (i.e., those not in viral capsids) [53]. All viral nucleic acids were then extracted using the NucliSENS easyMAG^®®^ bio-robot (bioMérieux).

#### 2.3.2. Reverse Transcription and Amplification

For each pool, the RNA virus-only and DNA virus-only libraries were constructed using a whole transcriptome (WTA) or a whole genome (WGA) amplification method, respectively, as previously described [41].

### 2.4. Next-Generation Sequencing

For each pool, 1 µg from each library was pooled together, whenever possible, to construct RNA plus DNA viral libraries. High-throughput sequencing was carried out at the genomics center of the Institut Pasteur, Paris. Shotgun libraries were prepared by standard Illumina protocols using 1 µg of total genomic DNA. Each sample (sera, kidney, and spleen) was tagged according to its provenance (species, organs, and habitats) using Illumina adaptor-specific primers. High-throughput shotgun metagenomic sequencing was carried out in two different sessions. The first round was completed using an Illumina MiSeq platform with 300-bp paired-end reads (eight samples, see Table 2). The second round was performed with an Illumina HiSeq 2500 platform with 250-bp paired-end reads (28 samples, see Table 2).

### 2.5. Bioinformatic Analyses

**Figure 1:**
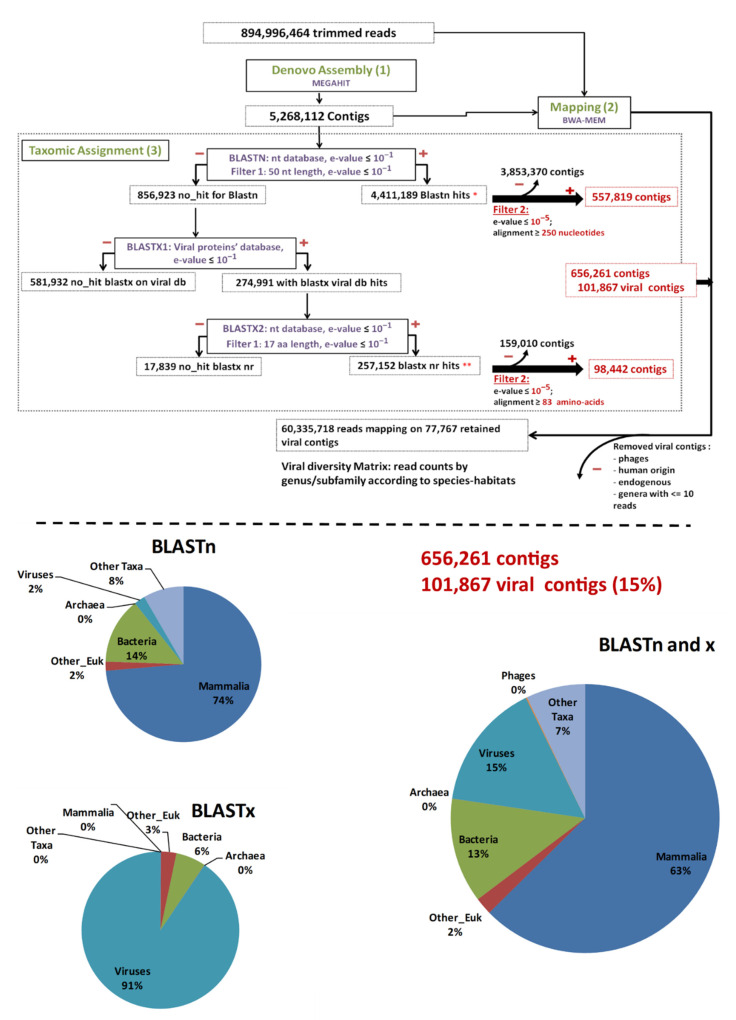
Main bioinformatic processing steps, data streams, and resulting taxonomic categories. “Other_Euk”: Other (non-mammalian eukaryotes).

Globally, after a first cleaning step, reads were assembled de novo (Step 1, Figure 1). Then, clean reads were mapped back to contigs in order to obtain the number of reads aligned to each contig (Step 2, Figure 1). The taxonomic assignment of contigs was achieved through BLASTn and BLASTx (Step 3, Figure 1). Finally, a matrix corresponding to the number of viral reads at the genus/subfamily level for each species–habitat was built for statistical analysis (Step 4, Figure 1).

All sequences were submitted to FaQCs [54] with an automated search for PhiX sequences, and quality filtering, after removal of adapters and poly-A tails (“*-phiX yes -adapter yes -polyA yes*”). The resulting clean sequence files were submitted to de novo assembly with MEGAHIT [55,56] using default parameters, which provide a set of k-mers from 21 to 141 with a step of 12 used in the assembly process, and the minimum contig length was set at 200 nucleotides (Step 1, Figure 1). Then cleaned reads were mapped back to the contigs using the BWA-MEM mapper [57] and Samtools [58] in order to obtain the number of reads for each contig (Step 2, Figure 1).

We used BLAST [59,60] for the taxonomic assignment of contigs (Step 3, Figure 1). All contigs were submitted to DISCONTIGOUS MEGABLAST BLASTn (e-value ≤ 10^−1^). Those without results in BLASTn were submitted to BLASTx. The BLASTx process comprised two steps: (1) using an in-house viral protein database, which was created by clustering (CD-HIT, 100% homology) the NCBI-nr database viral protein sequences (August 2018); (2) the set of positive contigs was subsequently submitted to BLASTx (e-value ≤ 10^−1^) against the whole NCBI-nr database. Both BLASTn and BLASTx results were filtered using in-house python scripts, which selected the best scoring match for each contig (max e-value of 10^−1^ and coverage of, respectively, >50 nucleotides and 17 amino acids in length for BLASTn and BLASTx) (Filter 1). Taxonomies were deduced from the BLAST results and fragments were assigned to selected viruses matching Taxids against the full name lineages file from NCBI (f) [61]. These two data sets were filtered again (Filter 2) according to the e-values and coverage according to both BLASTn and BLASTx (e-value = 10^−5^; coverage ≥ 250 nt or 83 amino acids in length) so as to consolidate the results. The remaining contigs were used for counting in the results. To provide a more accurate definition of the virus’s taxonomic status, each viral taxonomic identification was associated with host types, such as bacteria, vertebrates, invertebrates, amoeba, and plants, by consulting the ICTV [62], ViralZone, and virus host database websites [63,64].

As the presence of bacteriophages is unexpected in compartments such as the kidney, spleen, and blood, and it is difficult to attribute such bacteriophages to bacterial infections, contigs assigned to bacteriophages were discarded from the data set. In addition, a manual inspection was carried out and viruses known to be amplified in the laboratory (*Herpesviridae* and *Papillomaviridae*) and endogenous virus (*filovirus*) were discarded from the data set. Finally, based on mapping data, viral taxa (at the genus/subfamily level) identified with ≤10 reads were discarded from the data set in order to avoid the spurious presence of contigs due to potential contamination.

From these data, a data set associating the number of contigs with viral families was created. The number of viral families (categorized according to host type) associated with each species and its habitats was plotted and a heatmap was developed (Rstudio, “pheatmap” library). The heatmap represents the number of contigs associated with each viral family by species–habitats. The viral genomes’ completeness of assigned contigs was tested using CHECKV (version v0.7.0) and its associated database [65].

Lastly, a quantitative data set associating each contig with its number of reads was constructed with the number of reads associated with each taxonomic category at the subfamily or genus level.

The main steps are shown in solid-line boxes in green font characters, with the number in brackets representing the corresponding step in order of execution (de novo assembly, read mapping to contigs, and taxonomic assignment).

BLASTn and BLASTx, as sub-steps of taxonomic assignment, are shown in solid-line boxes with purple font characters. For each output, (+) and (−) stand for positive/kept and negative/discarded results, respectively, where they appear; taxonomic information acquisition and the filter-1 step are represented by red stars.

Subsequent filtering of contigs based on host (phages/human), endogenous status, and number of reads was carried out to obtain the final data set of both contigs and the corresponding reads matrix.

The taxonomic categories (Eukaryotes, Mammalia, Bacteria, Archaea, Viruses) to which contigs were assigned are shown as pie charts separately for BLASTn and BLASTx, and they were merged (BLASTn and x).

### 2.6. Statistical Analysis

To assess whether or not viral diversity is related to habitat type, diversity analyses were run for the four species present in at least two different habitats (pristine and disturbed forest *for P. guyannensis*, *P. cuvieri*, and *H. megacephalus*; disturbed forest, savannah, and peri-urban areas for *Z. brevicauda*) using the number of mapped reads on contigs assigned to each viral genus/subfamily (Supplemental data, Table S1).

As a prerequisite, before assessing local alpha diversities for the nine species–habitat combinations, we tested that (i) richness was not related to sequencing type using the Welch two-sample *t*-test); (ii) the number of viruses detected (genus/subfamily level) was not impacted by the number of individuals in each sequencing pool using a Pearson correlation test (R standard library) (Supplemental data, Table S2); (iii) each pool had been sufficiently sequenced to represent its diversity using rarefaction curves with the *rarefy* and *rarecurve* functions (Vegan R package) [66] with sampling at 231,725 reads (minimum number of viral reads across samples) and a step of 2000 reads (Supplemental data, Figure S1).

We then computed diversity indices for each of the nine species and habitat combinations and compared viral diversities, species by species, in their respective habitats. A synthesis between the most commonly accepted indices has been proposed [67] as a family of indices inspired in statistical physics [68], extending the link between diversity and entropy [69]. Based on α, related to Rényi’s entropy, H_α_ by N_α_ = exp(H_α_), three measures of diversity can be recovered: The total number of species (richness, α = 0), Shannon’s entropy (α = 1), and the inverse of Simpson’s dominance index (α = 2) [67,70]. The lower the α value is, the higher the weight given to rare species. The Hill α-diversity index values were generated with α in [0, 0.25, 0.5, 0.75, 1, 2], using the Vegan R package. A community A can be considered as more diverse than a community B if all Rényi’s entropy values for A are higher than for B when α runs over a given range (here 0 < α < 2) [71]. Therefore, to enhance comparisons between samples, we plotted Rényi’s entropy instead of Hill’s diversity for a given value of α followed by comparisons according to the parameters above [71].

Furthermore, to highlight the importance of the abundance of viral taxa (i.e., the number of reads per viral genus/subfamily) and to explore how rare species have contributed to diversity according to the habitat, we recomputed richness by progressive deletion of taxa with the lowest number of reads (i.e., setting their counts to 0 if they were under a defined threshold T). Thereafter, we quantified the impacts of these deletions on richness by calculating, for each case, the difference between the value of richness without deletion (R_0_,) and the richness value after suppression (R_T,_ richness value after deletion under T threshold): The higher the difference (R_0_–R_T_), the more numerous the rare species. The thresholds for deletion of a taxon were selected as fractions of the sample of the smallest size, i.e., fractions of 200,000 reads. We deleted all taxa with numbers of reads smaller than 0.01, 0.1, 1, 2, and 5% of this value, successively (i.e., 20, 200, 2000, 4000, 10,000) in all nine species–habitat combinations and calculated richness at each step.

### 2.7. Phylogenetic Analyses

Contigs assigned to four viral families (*Polyomaviridae*, *Flaviviridae*, *Togaviridae*, and *Phenuiviridae*) were selected from assembly files. Contigs were checked manually using the NCBI BLASTx web tool for the presence of stop codons. Then sequences presenting stop codons were deleted from further analysis. In order to infer phylogenetic relationships, we selected the closest sequences provided by BLAST during the taxonomic assignment process and some representatives of the family to be analyzed. In addition, for the *Polyomaviridae* and *Flaviviridae* analyses, sequences detected in rodents worldwide were also included. For the *Alphavirus* and *Phlebovirus* analysis, the data set including the closest sequences provided by BLAST was completed by sequences representative of the known antigenic complexes. All reference sequences were downloaded from the NCBI-nt database.

Accession numbers of viral sequences used to infer the phylogenetic trees are given in the respective phylogenetic reconstructions. After selecting the best-suited region for phylogenetic analyses, the Muscle algorithm [72] was used for multiple sequence alignments with default parameters. Pairwise sequence identity (at the nucleotide and amino acid levels) for each selected region were calculated using uncorrected p-distances. The best-fitted model of nucleotide or amino acid substitution for each analysis was selected using jModelTest 2 [73] and ProtTest 3 [74], respectively, under corrected Akaike information criteria (AICc). Bayesian phylogenetic analyses were performed using MrBayes 3.2 [75,76]. The Markov chain Monte Carlo (MCMC) algorithm was run with four chains with 2 million generations each, with trees sampled every 500 generations and a 25%burn-in. Validation of the inference was assessed based on the standard deviation of split frequencies, less than the expected threshold value of 0.01 in MrBayes and by inspecting the effective sampling size (ESS > 500) criterion in Tracer version 1.6 [77].

#### Nucleotide Sequence Accession Numbers

All virus sequences reported in this study were deposited in the GenBank nucleotide database under accession numbers MT732099 to MT732117. The data from Illumina sequencing were deposited in the GenBank Sequence Reads Archive under accession numbers SAMN15496919 to SAMN15496954.

## 3. Results

### 3.1. Illumina Sequencing and Bioinformatics Analyses

Overall, 453,865,988 paired-end raw reads (907,731,976 individual reads) were obtained that were 250–300 bp in length. After trimming by FaQcs, 96.62–99.56% of the reads were kept, totaling 894,996,464 reads.

These cleaned reads were assembled de novo for a total of 5,268,112 contigs using MEGAHIT (Figure 1 and Supplemental data, Table S3 for assembly statistics). The number of contigs ranged from 25,215 to 347,957. The mean number was 146,336 contigs/sample (Table 2). All these contigs were submitted to taxonomic assignment.

After megaBLASTn, 4,411,189 (83.73%) contigs were assigned to an organism, among which 50,431 were attributed to viruses. The remaining 856,923 (16.27%) unassigned contigs were first submitted to a BLASTx search against the in-house viral protein database (BLASTx1) (Figure 1). Only 274,991 contigs matched viral proteins, and they were all put through a second BLASTx step against the entire nr protein database (BLASTx2) (Figure 1). Overall, after this second BLASTx step (BLASTx2), 257,152 contigs were re-assigned to viruses, 3776 were assigned to other types of organisms, and 17,839 were not assigned at all (Figure 1).

To further avoid artifacts and false-positive results, the virus-assigned contigs were filtered at a coverage of ≥250 bp for BLASTn or ≥83 amino acids for BLASTx results and an e-value of ≤e−5 (Filter 2), resulting in 101,867 viral contigs, accounting for 1.93% of the total initial number of contigs and 15% of the assigned contigs (Figure 1).

Among the 101,867 viral contigs, 945 assigned to bacteriophages were discarded from further analyses. After a manual inspection, 20 sequences assigned to the *Herpesviridae* (human herpesviruses; >97% nucleotide identity), *Papillomaviridae* (human papillomaviruses; >90% nucleotide identity), and *Filoviridae* (endogenous) families were also removed (Figure 1). After removal of contigs with less than 11 reads mapped, 77,767 viral contigs were kept for further analyses. Among them, 118 contigs submitted to CHECKV were high quality, with completeness above 90%, and 72, 23, and 23 contigs were assigned to *Anelloviridae*, *Circoviridae*, and *Genomoviridae*, respectively.

### 3.2. Viral Diversity Detected through Species and Environments

**Figure 2:**
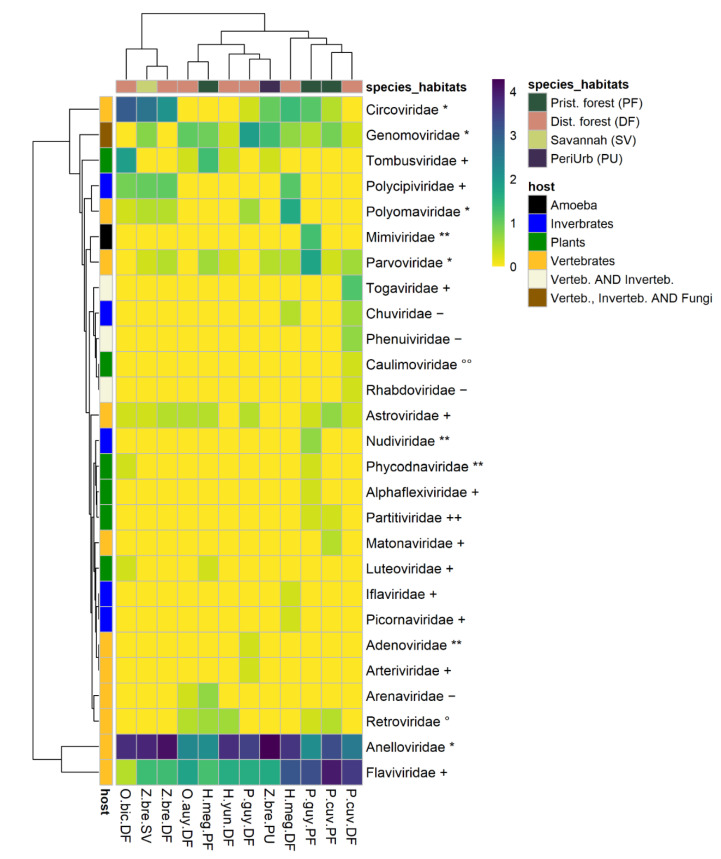
Heatmap of viral families’ numbers of contigs by species–habitat. Each cell representing a viral family in a species-habitat contains log (1 + N), (where N is the number of contigs assigned to a viral family in a species–habitat). The left row represents host-type (vertebrate, invertebrate, plants). Viral family names are marked with genome type, as follows: ** = dsDNA, * = ssDNA, **++** = dsRNA, **+** = ssRNA (+), **−** = ssRNA (−), **°°** = DNA-retrotranscribing, **°** = RNA-retrotranscribing. P.guy.PF: *Proechimys guyannensis* from pristine forest, P.guy.DF: *Proechimys guyannensis* from disturbed forest, P.cuv.PF: *Proechimys cuvieri* from pristine forest, P.cuv.DF: *Proechimys cuvieri* from disturbed forest, H.meg.PF: *Hylaeamys megacephalus* from pristine forest, H.meg.DF: *Hylaeamys megacephalus* from pristine forest, Z.bre.SV: *Zygodontomys brevicauda* from savannah, Z.bre.DF: *Zygodontomys brevicauda* from disturbed forest, Z.bre.PU: *Zygodontomys brevicauda* from peri-urban areas.

For the description of viral diversity, the results of viromes of different organs from a given species and in a given habitat were pooled together. The 77,767 viral contigs obtained were assigned to 27 families known to infect vertebrates, invertebrates, plants, and amoeba (Figure 2 and Table S4). Viral family presence varied largely across species–habitat categories. Indeed, we observed a pattern of ubiquity for some vertebrate viruses, along with those of the *Genomoviridae* family (which are commonly found in vertebrates, invertebrates, and fungi). On the other hand, some viruses, especially plant and invertebrate viruses, seemed more specific to the few species–habitat categories that they were found in. The patterns of ubiquity/specificity across species–habitat categories seemed to follow the patterns of the hosts (vertebrates, invertebrates, plants).

#### 3.2.1. Plant Viruses

Six plant-infecting viral families were detected from eight species–habitats, accounting for 49 contigs. Disturbed forest-originating samples from *P. guyannensis*, *H. megacephalus*, and *Z. brevicauda*, along with those from *Z. brevicauda* from savannah, contained no plant-infecting viruses. The most common plant-infecting viral family was the *Tombusviridae* family found in five species–habitats, followed by the *Luteoviridae*, *Partitiviridae*, and *Phycodnaviridae* families present in two species-habitats each. Lastly, *Alphaflexiviridae* and *Caulimoviridae* were all found in a single pool (Figure 2 and Table S2).

#### 3.2.2. Invertebrate Viruses

Five viral families of insect and invertebrate tropism were detected in six different species–habitats, totaling 106 contigs. The most common families were *Polycipiviridae* and *Chuviridae* present in four and two species–habitats, respectively. The remaining families (*Iflaviridae*, *Nudiviridae*, *Picornaviridae*) were detected in a single species–habitat (Figure 2 and Table S4). *P. cuvieri* from pristine forest; *P. guyannensis*, *O. auyantepui*, *H. yunganus*, and *H. megacephalus*, all from disturbed forest; and *Z. brevicauda* from peri-urban habitat contained no invertebrate viruses.

#### 3.2.3. Vertebrate Viruses

A total of 11 viral families strictly associated with vertebrates were detected, accounting for 77,438 contigs. ssDNA virus families such as *Anelloviridae*, *Circoviridae*, *Parvoviridae*, and *Polyomaviridae* were detected in 12, nine, eight, and five species–habitats, respectively. *Adenoviridae* (dsDNA virus)-assigned contigs were found only in *P. cuvieri* from disturbed forest. RNA viruses (*Riboviria*) accounted for six families. Several positive-sense RNA viral families were also detected: *Astroviridae*, *Arteriviridae*, *Flaviviridae* (*Hepacivirus*), and *Matonaviridae*. *Matonaviridae*-attributed sequences were found in only one species–habitat (*P. cuvieri* in pristine forest), just as *Arteriviridae* sequences were detected only in *P. guyannensis* from disturbed forest. By contrast, *Flaviviridae* (Hepacivirus) and *Astroviridae* had a greater presence across species–habitats, respectively, 12 of 12 and nine of 12 species–habitats. An *Arenaviridae*, an assigned sequence close to “Patawa virus” [46], was detected in *O. auyantepui* from disturbed forest (Figure 2 and Table S4).

#### 3.2.4. Potential Vector-Borne Viruses

Viral genera such as *Alphavirus* (*Togaviridae*) and *Phlebovirus* (*Phenuiviridae*), and the family *Rhabdoviridae*, are recognized as potential vector-borne viruses, since they infect both vertebrate and invertebrate hosts, were detected in one species–habitat. All contigs (19) attributed to vector-borne viruses were detected in *P. cuvieri* from disturbed forest (Figure 2 and Table S4).

### 3.3. Statistical and Ecological Analyses of the Viromes According to Hosts and Environments

#### 3.3.1. Sampling Effort on Viral Diversity

The Pearson correlation between the number of individuals per sample in a pool and the number of viral genera detected showed no significant association (*R*^2^ = −0.07, *p* = 0.694). The sequencing type had no impact on the viral diversity detected (two-sided unpaired Student’s *t*-test, *t* = −1.76, *p* = 0.105).

The rarefaction curves on the number of reads per sample, established for the nine species–habitat combinations, reached their asymptotes or started to plateau, suggesting that saturation was almost achieved if not in viral sequencing (Figure S1, Supplementary data). Hence, the sampling effort for our data set, both regarding the number of rodent individuals and the number of reads per sample, was adequate for diversity comparisons.

#### 3.3.2. Diversity across Species–Habitats

**Figure 3:**
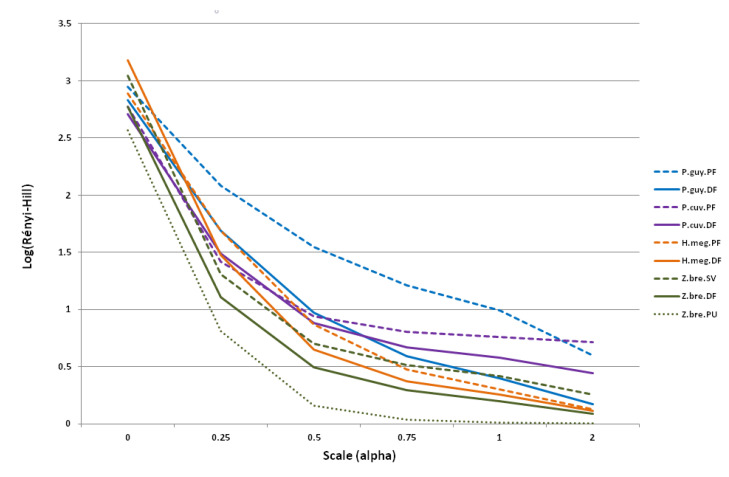
Ordination plot of Rényi’s entropy values by scale numbers (α = 0, 0.25, 0.5, 0.75, 1, and 2) for the four species present in different types of habitats (nine species–habitat combinations). Pristine forest and savannah are represented by dashed lines, disturbed forest by solid lines, and peri-urban areas by the dotted line. P.guy.PF: *Proechimys guyannensis* from pristine forest, P.guy.DF: *Proechimys guyannensis* from disturbed forest, P.cuv.PF: *Proechimys cuvieri* from pristine forest, P.cuv.DF: *Proechimys cuvieri* from disturbed forest, H.meg.PF: *Hylaeamys megacephalus* from pristine forest, H.meg.DF: *Hylaeamys megacephalus* from pristine forest, Z.bre.SV: *Zygodontomys brevicauda* from savannah, Z.bre.DF: *Zygodontomys brevicauda* from disturbed forest, Z.bre.PU: *Zygodontomys brevicauda* from peri-urban areas.

Viral richness oscillated between 10 for *Zygodontomys* in peri-urban habitats and 21 for *H. megacephalus* in disturbed forests (Table 3). On the other hand, Rényi’s entropy tended to converge between species and habitats for α = 2 (Figure 3). Most of the differentiation was between 0.5 < α < 1.0. Regarding the habitats of a given species, we observed that for *P. guyannensis* the diversity in pristine forest was higher than in disturbed forest (Figure 3). For *Z. brevicauda,* the diversity was higher in savannahs, followed by disturbed forests, and was lowest in peri-urban habitats. For *H. megacephalus* and *P. cuvieri*, the diversity was higher in disturbed forests for α < 0.25 and α < 0.75, respectively. For these species, diversity was higher in pristine forests for α > 0.25 and α > 0.75 (Figure 3). Thus, for two of the four rodent species trapped in two or more habitats, the diversity trend associated with different habitats was preserved over all α values between 0 (genera richness) and 2 (Simpson index).

**Figure 4:**
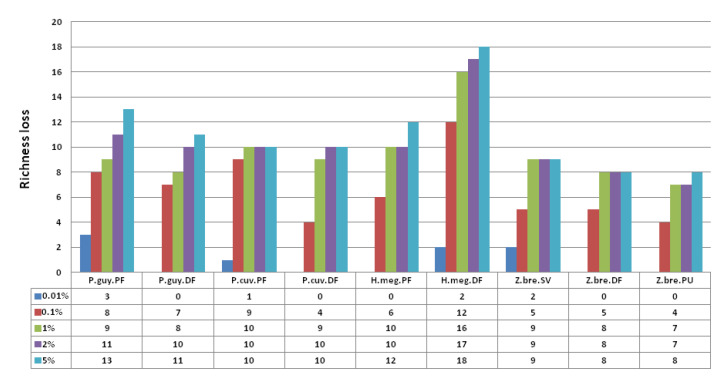
Bar plot of richness loss between original data set and different thresholds (minimum percent abundance) of suppressions by species–habitat combinations. Percent thresholds were 0.01, 0.1, 1, 2 and 5%. P.guy.PF: *Proechimys guyannensis* from pristine forest, P.guy.DF: *Proechimys guyannensis* from disturbed forest, P.cuv.PF: *Proechimys cuvieri* from pristine forest, P.cuv.DF: *Proechimys cuvieri* from disturbed forest, H.meg.PF: *Hylaeamys megacephalus* from pristine forest, H.meg.DF: *Hylaeamys megacephalus* from pristine forest, Z.bre.SV: *Zygodontomys brevicauda* from savannah, Z.bre.DF: *Zygodontomys brevicauda* from disturbed forest, Z.bre.PU: *Zygodontomys brevicauda* from peri-urban areas.

The richness loss, calculated for the nine species–habitat combinations with different removal thresholds of rare genera, is shown in Figure 4. *P. guyannensis* and *P. cuvieri* showed, at all threshold values, greater richness loss in pristine forest compared with disturbed forest, revealing that viromes from pristine habitats possessed higher numbers of rare viral entities (at the genus and subfamily levels according to their taxonomic classification) and fewer dominant ones than disturbed environments (Figure 4). *Z. brevicauda* showed higher richness loss in savannah compared with disturbed forest and peri-urban areas, showing that rare viral entities are more frequent in savannah than in the two other types of habitats. On the other hand, greater richness loss was observed for *H. megacephalus* in disturbed forest compared with pristine forest, highlighting the importance of rare entities in disturbed environments for this species (Figure 4). We observed that the hierarchy of richness loss between habitats was similar over all threshold values for each rodent species. Some of the differences in viral diversity between habitats for a given species are predominantly due to rare viral entities, rather than fractions of abundant ones.

### 3.4. Phylogenetic Relationships of Selected Viruses

For phylogenetic analyses, we chose viral families for their frequent presence in the samples (*Polyomaviridae* and *Flaviviridae*-*Hepacivirus*) and their interest as potential EID agents because they are arthropod-borne (*Phlebovirus* and *Alphavirus*).

#### 3.4.1. Rodent Polyomaviruses

The *Polyomaviridae* family is composed of four genera: *Alphapolyomavirus*, *Betapolyomavirus*, *Gammapolyomavirus*, and *Deltapolyomavirus* [78]. Each genome is composed of a circular dsDNA of approximately 5 kb. Polyomaviruses (PyVs) may be transmitted through either direct contact or by aerial or fecal–oral routes.

Overall, 54 contigs were assigned to the *Polyomaviridae* family and detected in four species: *O. bicolor*, *Z. brevicauda*, *H. megacephalus*, and *P. guyannensis* (Table S4). In kidney samples, one contig was detected in *O. bicolor* (disturbed forest); six were detected in *Z. brevicauda* (three from savannah and three from disturbed forest); 139 were detected in *H. megacephalus* (disturbed forest); and five were detected in *P. guyannensis* (disturbed forest). In spleen samples, two contigs were identified in *O. bicolor* (disturbed forest), two in *Z. brevicauda* (savannah and disturbed forest), and one in *H. megacephalus* (disturbed forest).

After alignment, the longest common sequences identified from the four species covered a fragment of 498 nucleotides of the Large T antigen (*LTAg*). Sequence fragments were named *O. bicolor polyomavirus 1* (*ObicPyV-1*, GenBank accession. number. MT732103), *Z. brevicauda polyomavirus 1* (*ZbrePyV-1*, GenBank accession number MT732104), *P. guyannensis polyomavirus 1* (*PguyPyV-1*, GenBank accession number MT732102), and *H. megacephalus polyomavirus 1* (*HmegPyV-1*, GenBank accession number MT732105).

The *ObicPyV-1* and *ZbrePyV-1* sequences shared 100% nucleotide and amino acid identity. *HmegPyV-1* displayed 99.22% nucleotide identity and 98.83% amino acid identity with the two previous sequences (Supplemental data, Table S5). *PguyPyV-1* displayed substantial divergence from those two sequences—50.80% nucleotide identity and 39.16% amino acid identity with the *ObicPyV-1* and *ZbrePyV-1* sequences—and showed 50% and 37.95% nucleotide identity and amino acid identity, respectively, with *HmegPyV-1*. Compared with other polyomavirus sequences, *ObicPyV-1* and *ZbrePyV-1* showed the highest percentages of identity (61.45% and 57.23% in nucleotide identity and amino acid identity, respectively) with *Sciurus carolinensis polyomavirus 1* (GenBank accession number MK671096); and *HmegPyV-1* showed 60.04% nucleotide identity and 54.02% amino acid identity with it (Supplemental data, Table S5). *PguyPyV-1* showed the highest level of nucleotide identity (67.78%) with *Pan troglodytes PyV3* (GenBank accession number YP_009094197) and 63.25% amino acid identity with *Merkel cell Polyomavirus* (GenBank accession number YP_009111421). *PguyPyV-1* also showed 59.44% nucleotide identity and 52.99% amino acid identity with the Alphapolyomavirus *Myocastor coypus polyomavirus 1* (GenBank accession number NC_040573) from a rodent species originating from South America.

**Figure 5:**
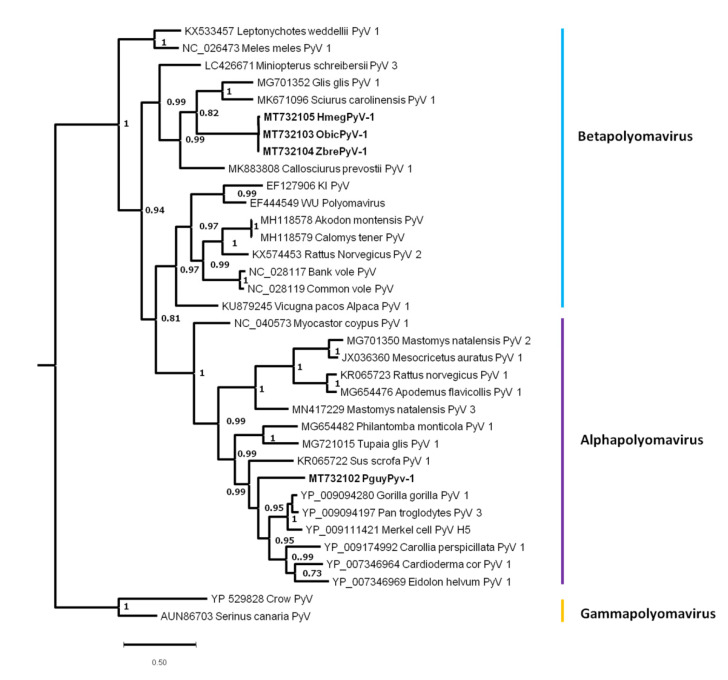
Phylogenetic analysis of partial sequences of the Large T antigen (LTAg) (alignment of 166 amino acid positions) of *Polyomaviridae* representatives (Alpha, Beta, and Gammapolyomaviruses with Gammapolyomaviruses as the outgroup). The tree was inferred from amino acid sequences using the Bayesian method with the WAG model. Sequence identifiers include the NCBI accession number and the isolate name. Posterior probabilities of the Bayesian analysis (>70%) are shown next to the nodes. The scale bar indicates amino acid substitutions per site. Sequences identified in this study are shown in bold.

The phylogenetic analysis carried out on 166 amino acid-long sequences of the *LTAg* identified the two monophyletic clades corresponding to the *Alphapolyomavirus* (posterior probability = 1) and *Gammapolyomavirus* (posterior probability = 1) genera, whereas *Betapolyomavirus* genus was not supported. *ObicPyV-1*, *ZbrePyV-1*, and *HmegPyV-1* polyomavirus sequences were clustered with polyomavirus sequences derived from Sciuridae (*Sciurus corolinensis)* and Gliridae (*Glis glis* and *Callosciurus prevostii*), a group of sequences that belongs to the *Betapolyomavirus* genus. These sequences are also associated with *Miniopterus schreibersii polyomavirus 3* (posterior probability = 0.99). Among alphapolyomaviruses, *PguyPyV-1* was not related to any other PyVs from rodents. It possessed a basal position of a clade consisting of viruses originating from primates and bats (posterior probability = 0.99) and to a lesser extent to PyVs from Artiodactyla and Scandentia (Figure 5).

#### 3.4.2. Rodent Hepaciviruses

Hepaciviruses (HVs) constitute a genus that belongs to the ssRNA+ family of *Flaviviridae*. Their genome is approximately 10 kb long and encodes a single ORF translated into a polyprotein, which is processed by viral and cellular proteases, giving mature proteins. According to Smith and colleagues [79], there are 14 HV species in mammals (HV-A to HV-N), among which six are hosted by rodents (HV-E to HV-J). The main mode of transmission of HVs is vertical, but the fecal route for cross-species transmissions has been suggested.

A total of 16,686 contigs were assigned to HVs (Table S4). These sequences were found in 18 of 36 pools (seven sera, six kidneys, and five spleens) and in all species included in the study. After re-examination of contigs and progressive multiple alignments, an *NS5B* (i.e., *RDRP*) fragment of 384 nucleotides was chosen for subsequent phylogenetic analyses. This region included the main known mammal HVs and rodent HVs (RHVs) identified in five of the seven species analyzed (i.e., *P. cuvieri* with four contigs, *P. guyannensis* with one contig, *Z. brevicauda* with two contigs, *H. megacephalus* with one contig, *H. yunganus* with three contigs). We attributed arbitrary names by contracting rodent species names with “HV” for Hepacivirus with an incremental number to differentiate sequences from the same host species.

The percentages of identity between HV sequences obtained in this study ranged from 36.67% to 97.58% in nucleotides and from 27.27% to 97.27% in amino acids, showing their great diversity (Supplemental data, Table S6). Briefly, PcuvHV4 (GenBank accession number MT732113), HmegHV1 (GenBank accession number MT732114), and HyunHV3 (GenBank accession number MT732116) sequences shared the highest percentages of identity (97.27–97.58% nucleotide identity and 94.55–96.36% amino acid identity) while PguyHV1 (GenBank accession number MT732106), PcuvHV1-3 (GenBank accession number MT732107–MT732109), ZbreHV1-2 (GenBank accession number MT732112, MT732112), and HyunHV1 (GenBank accession number MT732110), even if close, showed a greater divergence, with the percentage of identity ranging from 58.48% to 96.06% in nucleotides and from 64.55% to 97.27% in amino acids. These two groups of sequences were separated into two distinct clusters distantly related to each other. The HyunHV2 sequence (GenBank accession number MT732115) had the highest percentages of identity (64.24% in nucleotides and 71.82% in amino acids) with another Sigmodontinae rodent HV (*Oligoryzomys nigripes* Hepacivirus, accession number MH370348). Compared with the other RHVs, the first group of sequences (PcuvHV4, HmegHV1, and HyunHV3) showed 54.64–54.45% and 60–64.55% nucleotide identity and amino acid identity, respectively, with *Meriones meridianus* RHV (Supplemental data, Table S6). This group is also related to *Rhabdomys pumilio* RHV-I (GenBank accession number KC411806), showing between 58.48% and 59.09% nucleotide identity and between 52.72% and 56.36% amino acid identity. The second group showed 58.79–69.39% and 56.36–77.27% nucleotide identity and amino acid identity, respectively, with *Proechimys semispinosus* HV (GenBank accession number MG822666).

**Figure 6:**
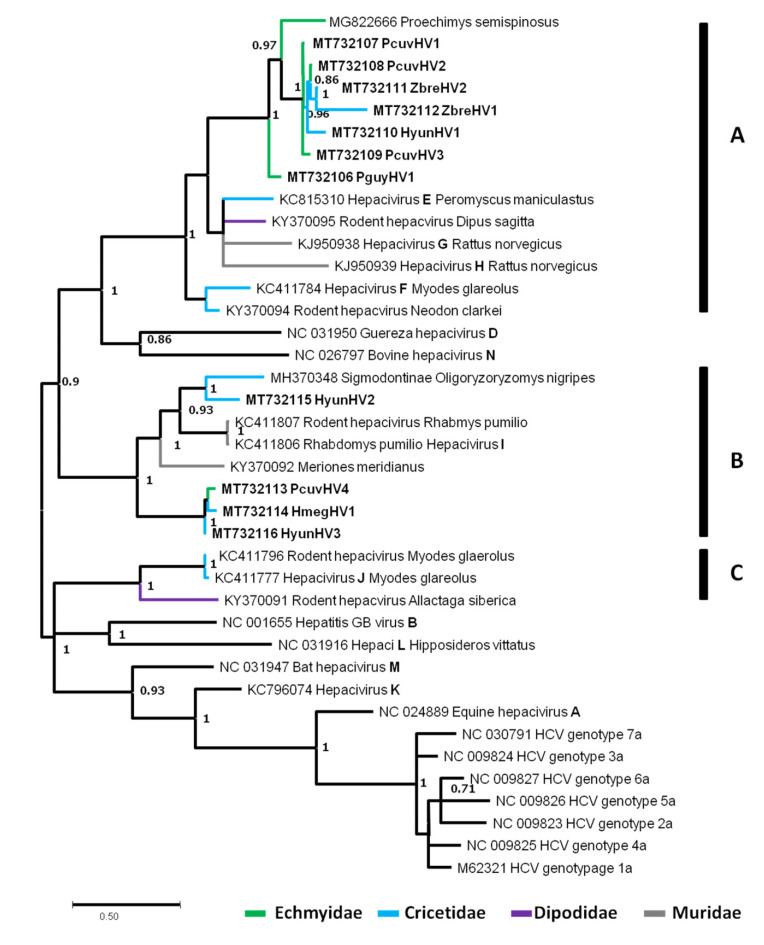
Unrooted phylogenetic tree based on the *ns5b* (alignment of 135 amino acid positions) of rodent hepaciviruses (RHVs). The tree was inferred from amino acid sequences using the Bayesian method with the WAG model. Sequence identifiers include the NCBI accession number and the isolate name. Posterior probabilities of the Bayesian analysis (>70%) are shown next to nodes. The scale bar indicates amino acid substitutions per site. The sequences identified in this study are shown in bold. The sequences identified in this study clustered in three well supported groups named **A**, **B** and **C** for ease of description.

Three groups (A, B, C) of sequences regrouping rodent HVs were identified, highly supported with posterior probabilities of 1 (Figure 6). Group A was subdivided into three subgroups. The first one comprised seven sequences identified here (PguyHV1, PcuvHV1-3, ZbreHV1-2, HyunHV1) and the previously identified RHV characterized in *P. semispinosus*. This subgroup was supported with a posterior probability of 1 and represented a group comprising Cricetidae and Echimyidae. It was related, however, with low support to a second subgroup composed of HV sequences identified in *Dipus sagitta*, *Peromyscus maniculatus*, and *Rattus norvegicus*, with these two last species hosting RHVs species E, G, and H. The third subgroup was composed two RHV sequences (*Neodon clarkei* HV and *Myodes glareolus* RHV-F). Group B contained the four remaining sequences from our samples (PcuvHV4, HmegHV1, HyunHV2-3), along with *Oligoryzomys*, *Meriones*, and *Rhabdomys* RHVs. PcuvHV4, HmegHV1, and HyunHV3 were grouped together (posterior probability = 1). The HyunHV2 sequence was clustered with the RHV sequence of *Oligoryzomys nigripes* (posterior probability = 1). This last group is related to RHV-I species hosted by *Rhabdomys pumilio* and to a lesser extent to RHV hosted by *Meriones meridianus*. Group C was composed of three RHVs sequences among which was the RHV-J species identified in *Myodes glareolus* (Figure 6). All these groups did not seem to follow a co-evolution model with their rodent hosts since HVs identified in different rodent families were found independently in all groups. In addition, for a given rodent species, diverse and distantly related HV sequences were identified.

#### 3.4.3. Alphaviruses (*Togaviridae*)

The *Togaviridae* family is composed of ssRNA+ viruses. Their genome, 10–12 kb in size, is composed of nonstructural and structural parts. The *Togaviridae* family has recently become a monogenus family exclusively composed of alphaviruses since its former sibling genus *Rubivirus* was removed to its own family (*Matonaviridae*). Alphaviruses are classified as antigenic complexes, such as VEE (Venezuelan equine encephalitis), EEE (Eastern equine encephalitis), WEE (Western equine encephalitis), etc. Alphaviruses are mainly transmitted by mosquitoes.

A total of 14 contigs were identified, all from *P. cuvieri* sera from disturbed forest. They covered 7682 nucleotides and both the structural and non-structural parts of the genome. We used the structural part of the genome (1989 nucleotides) for phylogenetic analyses including representatives of the main antigenic complexes (VEE, WEE, Semliki Forest, etc.). *P. cuvieri* VEE showed the highest levels of nucleotide and amino acid identity (94.83% and 98.69%, respectively) with the VEE strain Cabassou, a member of the antigenic complex of the same name (Supplemental data, Table S7).

**Figure 7:**
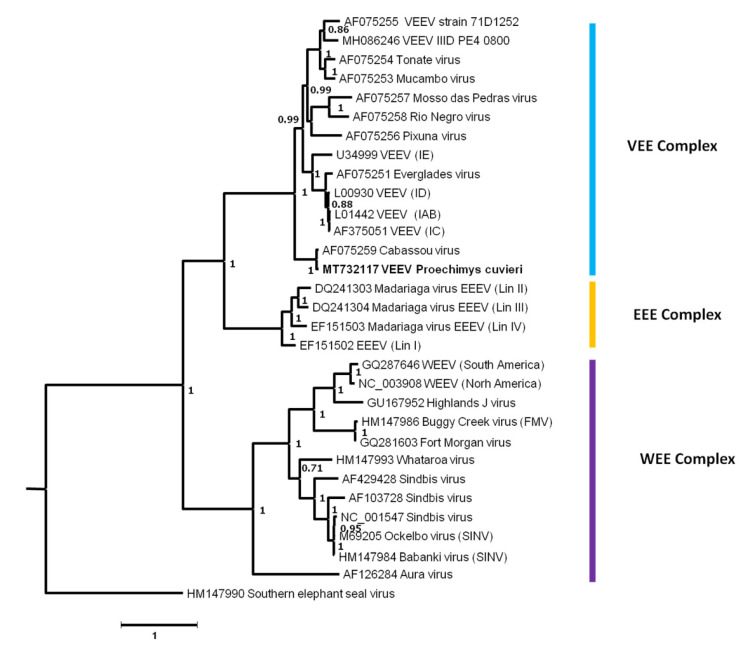
A phylogenetic tree based on one fragment of the structural protein (alignment of 1989 nucleotide positions) of each well-known representative of an alphavirus antigenic complex. The tree was inferred from nucleotide sequences using the Bayesian method with the GTR + G (general time reversible) model. Sequence identifiers include the NCBI accession number and the isolate name. The posterior probability of the Bayesian analysis (>70%) is shown next to each node. The scale bar indicates the (nucleotide) substitution rate. The sequence of *P. cuvieri* VEE is highlighted in bold and the main antigenic complexes are indicated.

The phylogenetic tree showed that *P. cuvieri* VEE clustered together with VEE Cabassou (posterior probability = 1), forming a clade with a basal position of the VEE complex. The clade was highly supported (posterior probability = 1) (Figure 7). The other major alphavirus complexes, EEE and WEE, were also supported with posterior probability values of 1 (Figure 7).

#### 3.4.4. Rodent Phleboviruses

Phleboviruses are members of the *Bunyavirales* order and belong to the *Phenuiviridae* family. They possess a segmented (three segments: small, medium, large) negative-sense ssRNA genome. Phleboviruses are arthropod-borne viruses frequently hosted by phlebotomin species (sandflies), but also by mosquitoes, ticks, and culicoides from which they are transmitted to humans and other vertebrates. Currently, 66 species have been recognized by the ICTV [80] and other species remain to be described. Phleboviruses have been classified in two antigenic groups including ten different species complexes [81,82].

Phlebovirus-attributed sequences were found in *P. cuvieri* samples from disturbed forest only, one from sera and three from spleen samples. The phylogenetic analysis was based on a 179-aa segment of the nucleocapsid. The *P cuvieri* phlebovirus sequence showed 79.80% and 95.78% nucleotide and amino acid identity, respectively, with Bujaru phlebovirus, which was identified from a *P. guyannensis* rodent in Brazil (Supplemental data, Table S8).

**Figure 8:**
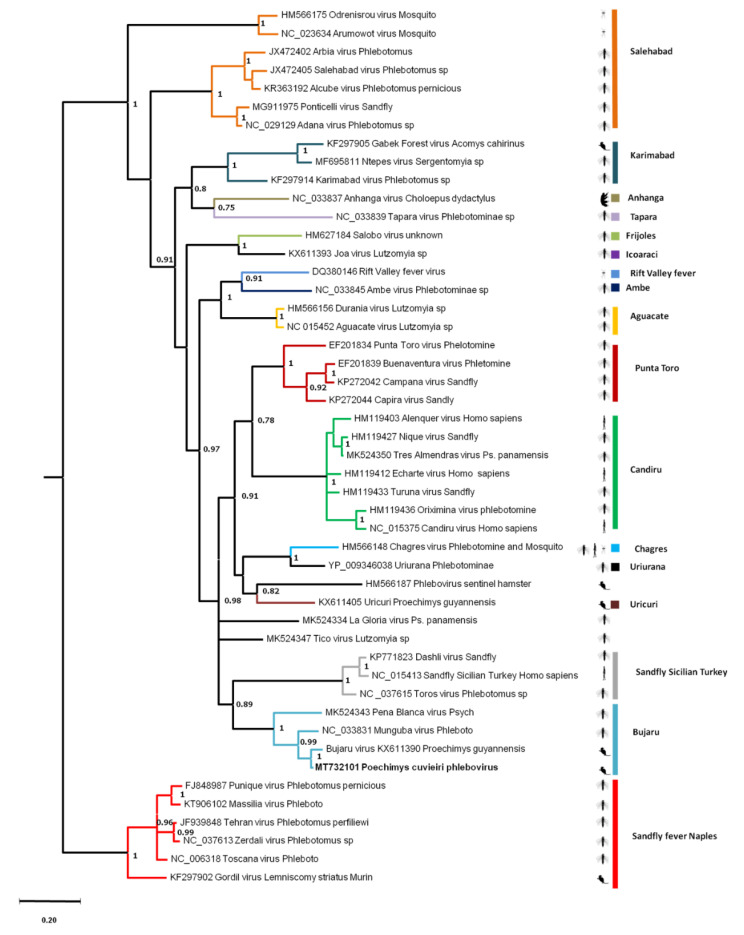
A phylogenetic tree based on one fragment of the nucleocapsid protein (alignment of 537 nucleotides/179 amino acid positions) of each well-known phlebovirus antigenic complex representative. The tree was inferred from amino acid sequences using the Bayesian method with the WAG model. Sequence identifiers include the NCBI accession number and the isolate name. Posterior probabilities of the Bayesian analysis (>70%) are shown next to the nodes. The scale bar indicates amino acid substitutions per site. The sequence of *P. cuvieri* phlebovirus is indicated in red. The species–complexes (established by ICTV or suggested in [81,82]) are indicated with colored bars (if many members are present) and dots (if one member is represented). Names of established species–complexes are indicated in bold and italics. Animal pictures were downloaded from phylopic web site (http://phylopic.org/) [83].

*P. cuvieri* phlebovirus were clustered phylogenetically with Bujaru virus with high support (posterior probability = 1). These *Proechimys*-originating sequences were grouped under the Bujaru serogroup with Munguba and Peña Blanca, both isolated from sandflies. The ancestral node of these four viruses was supported with a high posterior probability (pp = 1) (Figure 8). Nevertheless, phylogenetic relationships between all species–complexes remained unresolved with low support observed for basal nodes.

## 4. Discussion

Over the past decade, virome studies exploring the roles of wild species as reservoirs of infectious diseases have become more common thanks to the technological breakthrough of high-throughput sequencing. Considering that some species are reservoirs of numerous viruses, some of which have large impacts on human health, studies on viral diversity in rodents have recently increased [3,9,84,85,86]. Hence, 173 viral species belonging to more than 65 genera have been described in rodents to date, among which 53 are zoonotic, such as mammarenaviruses and hantaviruses [30,87]. However, few studies have explored the links among viral diversity, host ecology, and habitats [8,88,89]. Here, we presented the viral diversity identified in three different organs of seven rodent species from French Guiana, according to their natural hosts and habitats, and further explored the phylogenetic relationships of several viruses of interest for human health.

In order to ascertain the viral infection status in natural reservoirs and to identify a large number of vertebrate-related viruses, we chose to study three types of organs representing different tropisms of viruses. The kidney is the target organ of viruses that use the urinary tract to disseminate, such as hantavirus, arenaviruses, and paramyxoviruses, whereas viruses such as dengue or West-Nile have been detected in the spleen, a blood reservoir. Finally, serum is one of the most important media for the transmission of arboviruses. Furthermore, the analysis of such organ samples also limits the potential errors in the taxonomic assignment of new viruses compared to those that may be detected in respiratory or fecal samples. The latter viruses could indeed be from environment plants, insects, or fungi, and only incidentally found in rodents. Together, the use of organs should give a good representation of vertebrate viruses hosted by rodents [84].

Overall, this study identified 77,767 viral-associated contigs distributed within 27 viral families known to infect vertebrates, invertebrates, plants, and amoeba. The viromes were quantitatively dominated by vertebrate viral sequences (>99% of both contigs and reads were assigned to 11 viral families known to strictly infect vertebrates) and to a lesser extent to viral sequences from invertebrates, plants, and amoeba. Nevertheless, the smaller number of invertebrate and plant virus sequences indicates non-negligible diversity, accounting for 12 families.

The different viral families, whether originating from invertebrates, plants, or vertebrates, were not evenly distributed within the different species and habitats. Viruses from *Parvoviridae*, *Circoviridae*, *Astroviridae*, and *Anelloviridae* from vertebrates were found in most species and habitats and can be considered as generalists. These ubiquitous viruses were already reported in wild rodents in the United States where the *Circoviridae* family was the most abundant among the 24 families described [86], and in wild brown rats in Germany, with viruses of the *Parvoviridae* family [90]. On the other hand, viruses belonging to the *Caulimoviridae* (from plants), *Iflaviridae* (from invertebrates), or *Arteriviridae* (from vertebrates) families were rare and only present in some species and/or habitats. These differences in the distribution of viral families can be put in perspective by hypothesizing a rare biosphere for microbial communities in oceanic waters [91], with a portion of a few dominant microbial species and a second large, unexplored fraction with rare species. Accordingly, viromes in rodents could be dominated by a few dominant families, and a long distribution tail shaping a rare virosphere.

Such differences in virus abundance could be related to the ecology of the viruses (i.e., their ability to infect host cells and to persist and replicate) and to the ecology and behavior of their rodent hosts in a given habitat, such as a modified diet in a disturbed environment. The role of vectors in viral transmission and their diversity according to the environment can also have an impact on viral diversity. Indeed, for *P. cuvieri* and *H. megacephalus,* fourfold more viral families of invertebrate and vertebrate viruses have been detected in disturbed forest compared with pristine forest. In these two opportunistic species, diet can be supplemented by invertebrates when fruits and seeds are lacking [92], with subsequent impacts on their virome structures. On the other hand, a more specialized diet should restrict the range of viral diversity. Similar virome compositions were previously observed in house mice [3] and brown rats [85] in New York City, suggestive of an adaptive diet.

Viral diversity indices and the relative dominance levels of viral species were also impacted by the level of disturbance and the type of habitat. In this study, the highest viral diversity index values were mainly observed in pristine habitats where the highest diversity of hosts was also recorded. The viromes of *P. guyannensis* and *H. megacephalus* in pristine forest showed the highest diversities (mainly driven by viruses originating from plants) compared with their counterparts from disturbed forest. This trend was nevertheless not found in *P. cuvieri*, for which viral diversities were comparable between habitats (pristine vs. disturbed forest), but a higher number of rare viral entities were in pristine forest. In contrast, *H. megacephalus* presented a high number of rare viruses in disturbed forests. *Z. brevicauda*, the only species also sampled in the savannah, showed the highest viral diversities in this habitat, also reflecting the richness of the savannah ecosystem [93,94]. Peri-urban areas had the lowest viral diversity, which may be related to overall low biodiversity.

Among vertebrate hosts, rodents have been described as major reservoirs of arboviruses such as *Togaviridae*, *Flaviviridae*, and *Bunyaviridae* [95], and can serve as amplifiers of viruses that can be transmitted to humans. For instance, Cabassou virus (genus *Alphavirus*) was detected in *P. cuvieri* in disturbed forest. The circulation of arboviruses in disturbed habitats could be the result of increased contacts with vectors and may also reflect the lowest diversity of hosts available for arthropods to feed on.

The likelihood of disease emergence is indeed commonly accepted to increase in disturbed habitats [96]. The transmission of viruses from forest species to humans may result from two mechanisms. First, anthropic activities can increase contact between wildlife and humans and thereby the risk of infection [36] when humans enter slightly modified habitats and come into contact with a pristine viral cycle. Secondly, in more degraded forests, environmental changes may disrupt some ecological barriers and impact the structure and dynamics of rodent and arthropod communities, species richness, and ecological functions [97]. This may favor generalist over specialist species and ultimately the dominance of more synanthropic ones. Feeding networks between hosts and hematophagous vectors consequently change, influencing the transmission of viruses and potentially increasing cross-species transmission events.

From a theoretical point of view, the dilution effect hypothesis explores how the decrease of biodiversity may increase the amplification of zoonotic diseases. Briefly, the dilution effect proposes that a high diversity of putative hosts and vector species dilutes the more efficient carriers and amplifiers of viruses in a community of less efficient species, consequently reducing the circulation of the harmful ones and lowering the likelihood of infection [98]. The dilution effect may affect cycles involving a single animal host (i.e., reservoir) and those with two host compartments, i.e., reservoirs and vectors. In the latter case, a decrease in vertebrate diversity may concentrate blood meals taken by arthropods on a lower number of species, resulting in a higher viral circulation as soon as those resilient vertebrate species are also efficient carriers. The dilution effect can be suggested to illustrate the links between the diversity of rodent hosts and the spread of some zoonotic viruses. A higher probability of hepacivirus infection in *P. semispinosus* has been related to a loss of diversity in hosts due to land-use change [99]. Additionally, hantavirus outbreaks in the Americas are related to environmental disturbances that result in a decrease in specific richness of non-murine rodents and in the dominance of a few *Muridae* species known to be more efficient reservoirs [28,100]. In French Guiana, all known human hantavirus cases occurred in agricultural and peri-urban areas, where rodent diversity is much lower than in forest habitats [33], likely favoring hantavirus circulation in most efficient reservoirs.

In this study, 14 viral families from *Rodentia* were detected of the 31 currently described. We established the phylogenetic relationships of viral sequences related to four viral families known to infect vertebrates including arthropod-borne viruses (*Polyomaviridae*, *Flaviviridae*, *Togaviridae*, and *Phenuiviridae*). Even if the sequences obtained were incomplete, their analysis added knowledge on viral evolution among rodent species in South America, a group of species with very few data available to date.

Most viral sequences were related to sequences previously detected in rodents from multiple geographic areas (Africa, Asia, and North and South America), suggesting common evolutionary processes. Nevertheless, cross-species transmission and spill-over events were also detected, emphasizing the importance of these mechanisms in their evolution. These events took place early during the evolution of mammals or could be linked to recent interactions between sympatric species, as suggested for polyomaviruses. Indeed, PyVs have been described in a wide range of hosts, including mammals, birds, amphibians, reptiles, fish, and invertebrates. Some PyVs are pathogenic for humans and animals [78]. In rodents, 45 PyVs have been described from 11 species originating from Europe, Asia, and Africa [30]. In South America, betapolyomaviruses were recently described in two Sigmodontinae (Cricetidae) species (*Akodon montensis* and *Calomys tener*), and an alphapolyomavirus in a Myocastoridae species (*Myocastor coypus*) [101,102]. We identified four PyV sequences in four species (Figure 4). The three Sigmodontinae PyV sequences (*ObicPyV1*, *ZbrePyV1*, and *HmegPyV1*) belong to the *Betapolyomavirus* genus, and the Echimyidae PyV sequence (*PguyPyV1*) to *Alphapolyomavirus*. The sequence identified in *P. guyannensis* did not cluster with the other alphaPyVs sequences from rodents and showed a basal position to a clade constituted of PyVs detected in primates and Chiroptera, suggesting duplication events [103]. Polyomaviruses were initially considered to be host-specific, with codivergence and lineage duplication being the main drivers of their diversification [6,104,105,106]. PyV cross-species transmissions were also identified, but they do not seem to play a major role in the diversification processes of PyVs [105,106,107]. In the present study, a duplication event can be suggested to explain the position of *PguyPyV1*, and host-switching events could explain the closeness of PyVs detected in the Sigmodontinae subfamily. Indeed, the high PyV sequence identity values observed between *O. bicolor*, *Z. brevicauda*, and *H. megacephalus* may reflect a geographical signature related to the sympatry of these three taxonomically related species, favoring PyV host-switching events [101,106]. Further work is needed to confirm this hypothesis so as to ascertain whether the novel PyVs detected in the present study show evidence of host-switching in the Sigmodontinae subfamily, and whether PyV host-switching is more common in rodents than in other mammalian orders.

We also identified a large number of hepacivirus sequences (HVs) in the seven species, suggesting a high prevalence of HVs in neotropical rodents. The *hepacivirus* genus prototype is the human-infecting *hepatitis C virus* (HCV). After its identification at the end of the 1980s, HCV remained, along with *GB-virus B* (GBV-B), the only known HV for years. Homologues were then described within a wide range of hosts, such as horses, bats, rodents, cows, dogs, and even sharks, thanks to high-throughput sequencing and extensive investigations [4,9,108,109,110,111,112,113]. Like polyomaviruses, HVs are considered to co-evolve with their hosts [4]. Nevertheless, their descriptions in a wide range of species, and more particularly in primates and rodents (RHVs), have demonstrated that they do not fully follow a co-speciation pattern [114]. The phylogenetic analysis of HVs, including those detected in this study, revealed the presence of genetically distinct RHVs in *P. cuvieri*, *H. yunganus*, and *Z. brevicauda*, and of two distantly related RHVs in *P. guyannensis* and *H. megacephalus* (Figure 5). All RHV clades did not seem to co-evolve with their hosts, since RHVs identified in Muridae, Dipodidae, Echimyidae, and Cricetidae rodents were independently found in different clades. In addition, the identification of different RHVs in a single rodent species demonstrated the high level of genetic diversity among RHV species, reinforcing the idea that strict co-evolution is unlikely [114]. This high diversity reflects the idea that host shifts seem to be the main driver of RHV evolution [4,114,115]. The same evolutionary pattern was already suggested for RHVs from rodents in China [9]. Thus, the evolutionary history of the *Hepacivirus* genus remains to be deciphered along with the role of their rodent hosts as zoonotic transmitters, given their basal phylogenetic position in relation to other mammalian hepaciviruses [4,85,116].

Alphaviruses are arboviruses infecting a large number of vertebrates [5]. They are maintained in enzootic cycles involving arthropods as vectors and small mammals and/or birds as amplifier hosts. Occasionally, spill-overs into humans and domesticated animals lead to disease [117]. Among alphaviruses, VEEV have caused epizootics and epidemics in South America and the southern United States in the past few decades [118,119,120]. The VEEV complex is divided into six subtypes, some of which are known to cause diseases in humans and horses while others are enzootic, but can potentially be transmitted to humans [121,122,123]. In French Guiana, two viruses belonging to the VEEV complex were previously reported: *Cabassou virus* (CABV) isolated from *Culex portesi*, belonging to the subtype V, and *Tonate virus* (TONV), isolated from a bird and associated with the subtype IIIB [117,124]. Only a few cases of TONV infection were detected in French Guiana, Suriname, and North America, and most patients showed febrile illness. However, severe cases of encephalitis have been described [124,125]. To date, no human case of CABV infection has been reported.

Here, we identified sequences related to CABV (98.69% amino acid identity) in *P. cuvieri* samples in disturbed environments. Given that rodents belonging to the genera *Proechimys*, *Sigmodon*, *Oligoryzomys*, and *Oryzomys* have already been described as the main reservoirs for enzootic VEEV strains, CABV may circulate between mosquitoes and rodents, such as *Culex (melanoconion) portesi* (where it was previously isolated) and *Proechimys* [31,117,122,126,127]. VEEV strains are important candidates for future emergence in South America given that their reservoir hosts and vectors are facing an increasing number of anthropogenic disturbances. The potential circulation of CABV in the human population should be further investigated because its symptoms resemble those of dengue fever and it could therefore, be mis- or undiagnosed [128,129].

The *Phenuiviridae* family comprises 19 genera, including the *Phlebovirus* genus, which currently has ten recognized species [80] distributed worldwide. Among them, 14 phleboviruses were identified in rodents and 21 remain to be classified [30]. In South America, four species have been detected in rodents: *Icoaraci*, *Itaporanga*, *Jacunda* (Candiru complex), and *Bujaru*. The phylogenetic analysis of the sequence identified in *P. cuvieri* suggests that it belongs to the Bujaru complex composed of *Munguba*, *Peña blanca*, and Bujaru viruses [82]. Previous studies have already identified phleboviruses belonging to the Bujaru complex in *P. guyannensis* in Brazil and in sandflies (Phlebotominae spp.) [82,130], suggesting that viruses of the complex circulate in wild fauna and potentially in humans. Nevertheless, the pathogenicity of viruses of the Bujaru complex in humans is not known since most of the human cases described to date were due to viruses of the *Candiru* species complex. As for CABV, the fact that no human case has been reported for Bujaru viruses can be related to under-diagnosis given the similarity of the associated symptoms with other arboviral diseases [131,132]. Further studies are needed to investigate the ability of phlebotomine to spread the virus from their natural hosts and to clarify the real impact of phlebovirus infections on human health.

## 5. Conclusions

Only a few studies on the viral diversity in rodents have been conducted, even though they comprise the first order of mammals in terms of the number of species and are considered an important source of viral zoonotic pathogens. In addition, while Amazonia is considered a hotspot of diversity for hosts and pathogens [133], most virome studies have been conducted in Asia and North America. In French Guiana, north of the Amazonian region, the description of the virome of seven neotropical rodent species allowed us to identify a large number of new viruses, most of which correspond to vertebrate viruses. These findings extend our knowledge on the host range and evolution of these viruses. We identified previously known viruses belonging to *Togaviridae* and *Phenuiviridae* in the spiny rat *P. cuvieri*, highlighting its role in the maintenance and circulation of these arthropod-borne viruses in disturbed areas. Further research is needed to better understand the transmission cycles and the ecology of the hosts and vectors involved. In addition, we showed that the diversity of rodent viromes varies according to the types of habitat, with higher viral diversity in pristine forests compared with disturbed forests for most rodent species. As well as the environment, the significance of species characteristics (including distribution, ecology, demography, and phylogenetic relationships), the importance of host switch throughout virus evolution, and the potential for local cross-species transmission should be studied to gain a better understanding of how viral diversity is shaped.

Environmental pressures on wild animal populations continue to grow, leading to increasing risks of contact between human and rodent populations. This could favor the emergence or re-emergence of viral diseases, including from viruses yet unknown or with undocumented roles on human health.

## Figures and Tables

**Table 1:** Sampling data of individuals by organ, species, and habitat. The composition of each pool, including organ, species, habitat, and number of individuals, is given. The last column corresponds to the total number of different individuals used to constitute each pool of each species in a given habitat. The last row corresponds to the totals of organs and individual samples for this study.

Pool Number	Sample ID	Organ	Species	Habitat	Nb. Individuals	Total of Different Individuals
1	k_Pguy_PF	Kidney	*Proechimys guyannensis*	Pristine Forest	5	12
2	r_Pguy_PF	Spleen	*Proechimys guyannensis*	Pristine Forest	8	
3	s_Pguy_PF	Sera	*Proechimys guyannensis*	Pristine Forest	12	
4	k_Pguy_DF	Kidney	*Proechimys guyannensis*	Disturbed Forest	24	29
5	r_Pguy_DF	Spleen	*Proechimys guyannensis*	Disturbed Forest	27	
6	s_Pguy_DF	Sera	*Proechimys guyannensis*	Disturbed Forest	27	
7	k_Pcuv_PF	Kidney	*Proechimys cuvieri*	Pristine Forest	9	35
8	r_Pcuv_PF	Spleen	*Proechimys cuvieri*	Pristine Forest	24	
9	s_Pcuv_PF	Sera	*Proechimys cuvieri*	Pristine Forest	31	
10	k_Pcuv_DF	Kidney	*Proechimys cuvieri*	Disturbed Forest	18	18
11	r_Pcuv_DF	Spleen	*Proechimys cuvieri*	Disturbed Forest	18	
12	s_Pcuv_DF	Sera	*Proechimys cuvieri*	Disturbed Forest	15	
13	k_Oauy_DF	Kidney	*Oecomys auyantepui*	Disturbed Forest	5	14
14	r_Oauy_DF	Spleen	*Oecomys auyantepui*	Disturbed Forest	11	
15	s_Oauy_DF	Sera	*Oecomys auyantepui*	Disturbed Forest	13	
16	k_Obic_DF	Kidney	*Oecomys bicolor*	Disturbed Forest	13	15
17	r_Obic_DF	Spleen	*Oecomys bicolor*	Disturbed Forest	14	
18	s_Obic_DF	Sera	*Oecomys bicolor*	Disturbed Forest	12	
19	k_Hyun_DF	Kidney	*Hylaeamys yunganus*	Disturbed Forest	11	11
20	r_Hyun_DF	Spleen	*Hylaeamys yunganus*	Disturbed Forest	11	
21	s_Hyun_DF	Sera	*Hylaeamys yunganus*	Disturbed Forest	10	
22	k_Hmeg_PF	Kidney	*Hylaeamys megacephalus*	Pristine Forest	2	5
23	r_Hmeg_PF	Spleen	*Hylaeamys megacephalus*	Pristine Forest	4	
24	s_Hmeg_PF	Sera	*Hylaeamys megacephalus*	Pristine Forest	3	
25	k_Hmeg_DF	Kidney	*Hylaeamys megacephalus*	Disturbed Forest	4	5
26	r_Hmeg_DF	Spleen	*Hylaeamys megacephalus*	Disturbed Forest	4	
27	s_Hmeg_DF	Sera	*Hylaeamys megacephalus*	Disturbed Forest	4	
28	k_Zbre_SV	Kidney	*Zygodontomys brevicauda*	Savannah	6	12
29	r_Zbre_SV	Spleen	*Zygodontomys brevicauda*	Savannah	6	
30	s_Zbre_SV	Sera	*Zygodontomys brevicauda*	Savannah	8	
31	k_Zbre_DF	Kidney	*Zygodontomys brevicauda*	Disturbed Forest	7	10
32	r_Zbre_DF	Spleen	*Zygodontomys brevicauda*	Disturbed Forest	7	
33	s_Zbre_DF	Sera	*Zygodontomys brevicauda*	Disturbed Forest	10	
34	k_Zbre_PU	Kidney	*Zygodontomys brevicauda*	Peri-urban	20	21
35	r_Zbre_PU	Spleen	*Zygodontomys brevicauda*	Peri-urban	20	
36	s_Zbre_PU	Sera	*Zygodontomys brevicauda*	Peri-urban	19	
	Totals	-	*-*	-	442	187

**Table 2:** Sequencing and bioinformatic processing data by organ, species, and habitat. It shows the number of raw reads, the corresponding Illumina Platform, the percentage of reads kept after trimming and cleansing, the number of assembled contigs, the percentage of cleaned reads mapped back to contigs, and the number of viral contigs.

Sample ID	No. of Raw Reads	Sequencing Type	% Cleaned Reads	No. of Contigs	% Mapped Reads	No. of Viral Contigs
k_Pguy_PF	39,505,174	Hiseq2500	98.12	323,113	97.81	101
k_Pguy_DF	24,142,444	Hiseq2500	98.99	143,955	97.88	161
k_Pcuv_PF	39,878,026	Hiseq2500	98.15	267,539	98.33	398
k_Pcuv_DF	32,782,446	Hiseq2500	99.56	88,325	99.30	4783
k_Oauy_DF	43,211,478	Hiseq2500	98.01	225,793	98.36	86
k_Obic_DF	3,999,474	MiSeq	97.55	31,337	98.92	2208
k_Hmeg_PF	35,419,066	Hiseq2500	98.50	285,067	97.96	71
k_Hmeg_DF	6,259,058	MiSeq	96.62	53,664	98.99	2998
k_Hyun_DF	31,133,790	Hiseq2500	98.48	159,779	98.77	142
k_Zbre_PU	34,912,952	Hiseq2500	98.48	181,251	98.79	150
k_Zbre_DF	6,754,874	MiSeq	98.35	56,113	98.75	1698
k_Zbre_SV	4,395,890	MiSeq	98.16	25,215	99.25	1656
r_Pguy_PF	40,349,120	Hiseq2500	98.01	330,777	97.87	79
r_Pguy_DF	29,988,950	Hiseq2500	98.22	231,108	97.69	125
r_Pcuv_PF	16,840,948	Hiseq2500	98.77	124,874	97.58	384
r_Pcuv_DF	27,294,898	Hiseq2500	98.28	267,976	97.05	909
r_Oauy_DF	33,188,412	Hiseq2500	98.57	184,913	98.19	109
r_Obic_DF	5,964,414	MiSeq	98.74	67,976	98.40	3506
r_Hmeg_PF	35,586,626	Hiseq2500	98.22	282,402	98.10	121
r_Hmeg_DF	7,633,778	MiSeq	97.32	53,117	99.38	1109
r_Hyun_DF	33,617,438	Hiseq2500	98.54	159,882	98.40	317
r_Zbre_PU	44,909,868	Hiseq2500	99.02	176,663	99.18	13,343
r_Zbre_DF	5,029,582	MiSeq	97.65	59,922	98.53	2591
r_Zbre_SV	4,296,504	MiSeq	95.59	79,251	97.40	617
s_Pguy_PF	20,326,680	Hiseq2500	98.86	56,461	98.83	2115
s_Pguy_DF	25,433,042	Hiseq2500	99.49	54,835	99.01	3473
s_Pcuv_PF	30,224,896	Hiseq2500	98.99	86,555	99.30	12,162
s_Pcuv_DF	25,361,476	Hiseq2500	98.15	347,957	96.80	3341
s_Oauy_DF	18,055,176	Hiseq2500	98.00	71,707	98.84	255
s_Obic_DF	26,895,348	Hiseq2500	99.23	103,205	98.55	2114
s_Hmeg_PF	32,497,436	Hiseq2500	99.48	75,533	99.19	248
s_Hmeg_DF	22,859,508	Hiseq2500	98.62	70,539	99.04	1862
s_Hyun_DF	24,873,840	Hiseq2500	98.78	258,141	96.09	6109
s_Zbre_PU	26,110,716	Hiseq2500	99.35	87,791	98.60	10,330
s_Zbre_DF	37,050,082	Hiseq2500	99.09	103,974	99.07	13,622
s_Zbre_SV	30,948,566	Hiseq2500	98.94	91,402	98.89	8574
Total	907,731,976	-	-	5,268,112	-	101,867

**Table 3:** Richness and Rényi’s entropy values for different values of α between 0 and 2 for the nine species–habitat combinations.

	P.guy.PF	P.guy.DF	P.cuv.PF	P.cuv.DF	H.meg.PF	H.meg.DF	Z.bre.SV	Z.bre.DF	Z.bre.PU
Richness	17	16	14	12	13	21	12	11	10
Renyi (α = 0)	2.94	2.83	2.77	2.71	2.89	3.18	3.04	2.77	2.56
Renyi (α = 0.25)	2.08	1.69	1.42	1.49	1.69	1.47	1.31	1.11	0.81
Renyi (α = 0.5)	1.55	0.97	0.94	0.89	0.87	0.65	0.70	0.49	0.16
Renyi (α = 0.75)	1.21	0.59	0.81	0.67	0.48	0.37	0.51	0.29	0.04
Renyi (α = 1)	0.99	0.40	0.76	0.58	0.30	0.25	0.42	0.20	0.01
Renyi (α = 2)	0.60	0.17	0.72	0.44	0.13	0.12	0.25	0.09	0.00

## Data Availability

All in-house scripts used in this study are available at the github repository under the link: github.com/stirera/rodentsvirome_filter1 (accessed on 9 August 2021).

## Supplementary Materials

The following are available online at https://www.mdpi.com/article/10.3390/v13091690/s1, Figure S1: Rarefaction curves for the four species present in different types of habitats (nine species–habitat combinations), Table S1: Number of viral read of each viral genus/subfamily according rodent species and habitats, Table S2: Sampling variables and viral richness for the nine species-habitat at organ level (Organ level pool name, Number of rodents individuals, Number of viral genera/subfamilies detected, Sequencing platform, Organ type), Table S3: Assembly statistics of each sample by organ, species- and habitat, Table S4: Number of viral contigs by rodent species/habitats and viral families, Tables S5–S8: Pairwise sequence identities precents in nucleotides and amino-acids for respectively partial *LTAg* (*Polyomaviridae*), partial *ns5b* (*Flaviviridae, Hepacivirus*), partial *Structural protein* (*Togavirida*) and *nucleocapsid protein* (*Phenuiviridae*; *Phlebovirus*).

## References

[1] Sanjuán R., Nebot M.R., Chirico N., Mansky L.M., Belshaw R. (2010). Viral mutation rates. J. Virol..

[2] Houldcroft C.J., Beale M.A., Breuer J. (2017). Clinical and biological insights from viral genome sequencing. Nat. Rev. Microbiol..

[3] Williams S.H., Che X., Garcia J.A., Klena J.D., Lee B., Muller D., Ulrich W., Corrigan R.M., Nichol S., Jain K. (2018). Viral diversity of house mice in New York City. MBio.

[4] Hartlage A.S., Cullen J.M., Kapoor A. (2016). The strange, expanding World of animal Hepaciviruses. Annu. Rev. Virol..

[5] Shi M., Lin X.D., Chen X., Tian J.H., Chen L.J., Li K., Wang W., Eden J.S., Shen J.J., Liu L. (2018). The evolutionary history of vertebrate RNA viruses. Nature.

[6] Buck C.B., Van Doorslaer K., Peretti A., Geoghegan E.M., Tisza M.J., An P., Katz J.P., Pipas J.M., McBride A.A., Camus A.C. (2016). The ancient evolutionary history of Polyomaviruses. PLoS Pathog..

[7] Roux S., Brum J.R., Dutilh B.E., Sunagawa S., Duhaime M.B., Loy A., Poulos B.T., Solonenko N., Lara E., Poulain J. (2016). Ecogenomics and potential biogeochemical impacts of globally abundant ocean viruses. Nature.

[8] Bergner L.M., Orton R.J., Benavides J.A., Becker D.J., Tello C., Biek R., Streicker D.G. (2020). Demographic and environmental drivers of metagenomic viral diversity in vampire bats. Mol. Ecol..

[9] Wu Z., Lu L., Du J., Yang L., Ren X., Liu B., Jiang J., Yang J., Dong J., Sun L. (2018). Comparative analysis of rodent and small mammal viromes to better understand the wildlife origin of emerging infectious diseases. Microbiome.

[10] Cobián Güemes A.G., Youle M., Cantú V.A., Felts B., Nulton J., Rohwer F. (2016). Viruses as winners in the game of life. Annu. Rev. Virol..

[11] Kreuder Johnson C., Hitchens P.L., Smiley Evans T., Goldstein T., Thomas K., Clements A., Joly D.O., Wolfe N.D., Daszak P., Karesh W.B. (2015). Spillover and pandemic properties of zoonotic viruses with high host plasticity. Sci. Rep..

[12] Dennehy J.J. (2017). Evolutionary ecology of virus emergence. Ann. N. Y. Acad. Sci..

[13] McMahon B.J., Morand S., Gray J.S. (2018). Ecosystem change and zoonoses in the Anthropocene. Zoonoses Public Health.

[14] Jones K.E., Patel N.G., Levy M.A., Storeygard A., Balk D., Gittleman J.L., Daszak P. (2008). Global trends in emerging infectious diseases. Nature.

[15] Siu A., Wong Y.C.R. (2004). Economic Impact of SARS: The case of Hong Kong. Asian Econ. Pap..

[16] Huber C., Finelli L., Stevens W. (2018). The economic and social burden of the 2014 Ebola outbreak in West Africa. J. Infect. Dis..

[17] European Centre for Disease Prevention and Control COVID-19 Situation Dashboard. https://qap.ecdc.europa.eu/public/extensions/COVID-19/COVID-19.html#global-overview-tabes.

[18] Bogich T.L., Chunara R., Scales D., Chan E., Pinheiro L.C., Chmura A.A., Carroll D., Daszak P., Brownstein J.S. (2012). Preventing pandemics via international development: A systems approach. PLoS Med..

[19] PREDICT. https://ohi.vetmed.ucdavis.edu/programs-projects/predict-project/about.

[20] Daszak P., Carroll D., Wolfe N., Mazet J. (2016). The global virome project. Int. J. Infect. Dis..

[21] Carroll D., Daszak P., Wolfe N.D., Gao G.F., Morel C.M., Morzaria S., Pablos-Méndez A., Tomori O., Mazet J.A.K. (2018). The Global Virome Project. Science (80).

[22] Kruse H., Kirkemo A.M., Handeland K. (2004). Wildlife as source of zoonotic infections. Emerg. Infect. Dis..

[23] Mollentze N., Streicker D.G. (2020). Viral zoonotic risk is homogenous among taxonomic orders of mammalian and avian reservoir hosts. Proc. Natl. Acad. Sci. USA.

[24] Corbet G.B., Hill J.E., Wilson D.E., Reeder D.M. (1993). Mammal Species Of the World: A Taxonomic and Geographic Reference.

[25] Huchon D., Douzery E.J.P. (2001). From the Old World to the New World: A molecular chronicle of the phylogeny and biogeography of hystricognath rodents. Mol. Phylogenetics Evol..

[26] Gravinatti M.L., Barbosa C.M., Soares R.M., Gregori F. (2020). Synanthropic rodents as virus reservoirs and transmitters. Rev. Soc. Bras. Med. Trop..

[27] Han B.A., Schmidt J.P., Bowden S.E., Drake J.M. (2015). Rodent reservoirs of future zoonotic diseases. Proc. Natl. Acad. Sci. USA.

[28] Ruedas L.A., Salazar-Bravo J., Tinnin D.S., Armién B., Cáceres L., García A., Díaz M.A., Gracia F., Suzán G., Peters C.J. (2004). Community ecology of small mammal populations in Panamá following an outbreak of Hantavirus pulmonary syndrome. J. Vector Ecol..

[29] Chen L., Liu B., Wu Z., Jin Q., Yang J. (2017). DRodVir: A resource for exploring the virome diversity in rodents. J. Genet. Genomics.

[30] Database of Rodent-Associated Viruses. http://www.mgc.ac.cn/DRodVir/.

[31] Meerburg B.G., Singleton G.R., Kijlstra A. (2009). Rodent-borne diseases and their risks for public health Rodent. Crit. Rev. Microbiol..

[32] De Thoisy B., Matheus S., Catzeflis F., Clément L., Barrioz S., Guidez A., Donato D., Cornu J.F., Brunaux O., Guitet S. (2014). Maripa Hantavirus in French Guiana: Phylogenetic position and predicted spatial distribution of rodent hosts. Am. J. Trop. Med. Hyg..

[33] Lavergne A., Matheus S., Catzeflis F., Donato D., Lacoste V., de Thoisy B. (2017). Rodent-borne viruses in French Guiana. Virologie.

[34] Liu J., Liu D.Y., Chen W., Li J.L., Luo F., Li Q., Ling J.X., Liu Y.Y., Xiong H.R., Ding X.H. (2012). Genetic analysis of hantaviruses and their rodent hosts in central-south China. Virus Res..

[35] Monath T.P., Newhouse V.F., Kemp G.E., Setzer H.W., Cacciapuoti A. (1974). Lassa virus isolation from *Mastomys natalensis* rodents during an epidemic in Sierra Leone. Science.

[36] Guégan J.-F.F., Ayouba A., Cappelle J., de Thoisy B. (2020). Forests and emerging infectious diseases: Unleashing the beast within. Environ. Res. Lett..

[37] Gentry A.H. (1988). Tree species richness of upper Amazonian forests. Proc. Natl. Acad. Sci. USA.

[38] Ellwanger J.H., Kulmann-Leal B., Kaminski V.L., Valverde-Villegas J.M., DA VEIGA A.B.G., Spilki F.R., Fearnside P.M., Caesar L., Giatti L.L., Wallau G.L. (2020). Beyond diversity loss and climate change: Impacts of Amazon deforestation on infectious diseases and public health. Anais da Academia Brasileira de Ciências.

[39] Han B.A., Kramer A.M., Drake J.M. (2016). Global patterns of zoonotic disease in mammals. Trends Parasitol..

[40] Allen T., Murray K.A., Zambrana-Torrelio C., Morse S.S., Rondinini C., Di Marco M., Breit N., Olival K.J., Daszak P. (2017). Global hotspots and correlates of emerging zoonotic diseases. Nat. Commun..

[41] Salmier A., Tirera S., de Thoisy B., Franc A., Darcissac E., Donato D., Bouchier C., Lacoste V., Lavergne A. (2017). Virome analysis of two sympatric bat species (*Desmodus rotundus* and *Molossus molossus*) in French Guiana. PLoS ONE.

[42] Bolatti E.M., Zorec T.M., Montani E., Hošnjak L., Chouhy D., Casal P.E., Barquez M. (2020). American Free-Tailed Bats (*Tadarida brasiliensis*) and identification of two novel mammalian viruses. Viruses.

[43] Matheus S., Lavergne A., de Thoisy B., Dussart P., Lacoste V. (2012). Complete genome sequence of a novel Hantavirus variant of Rio Mamore Virus, Maripa Virus, from French Guiana. J. Virol..

[44] Matheus S., Djossou F., Moua D., Bourbigot A.M., Hommel D., Lacoste V., Dussart P., Lavergne A. (2010). Hantavirus pulmonary syndrome, French Guiana. Emerg. Infect. Dis..

[45] Matheus S., Kallel H., Mayence C., Bremand L., Houcke S., Rousset D., Lacoste V., de Thoisy B., Hommel D., Lavergne A. (2017). Hantavirus pulmonary syndrome caused by Maripa virus in French Guiana, 2008–2016. Emerg. Infect. Dis..

[46] Lavergne A., de Thoisy B., Donato D., Guidez A., Matheus S., Catzeflis F., Lacoste V. (2015). Patawa Virus, a new Arenavirus hosted by forest rodents in French Guiana. Ecohealth.

[47] Lavergne A., de Thoisy B., Tirera S., Donato D., Bouchier C., Catzeflis F., Lacoste V. (2016). Identification of lymphocytic choriomeningitis mammarenavirus in house mouse (*Mus musculus*, Rodentia) in French Guiana. Infect. Genet. Evol..

[48] Sikes R.S. (2016). 2016 Guidelines of the American Society of Mammalogists for the use of wild mammals in research and education. J. Mammal..

[49] Direction régionale de l’ONF Guyane (2017). Occupation du sol en 2015 sur la Bande Littorale de la Guyane et son Évolution Entre 2005 et 2015.

[50] European Environment Agency (1994). European Environment Agency, Copenhagen Corine Land Cover.

[51] Ivanova N.V., Dewaard J.R., Hebert P.D.N. (2006). An inexpensive, automation-friendly protocol for recovering high-quality DNA. Mol. Ecol. Notes.

[52] Borisenko A.V., Lim B.K., Ivanova N.V., Hanner R.H., Hebert P.D.N. (2008). DNA barcoding in surveys of small mammal communities: A field study in Suriname. Mol. Ecol. Resour..

[53] Allander T., Emerson S.U., Engle R.E., Purcell R.H., Bukh J. (2001). A virus discovery method incorporating DNase treatment and its application to the identification of two bovine parvovirus species. Proc. Natl. Acad. Sci. USA.

[54] Lo C.-C., Chain P.S.G. (2014). Rapid evaluation and quality control of next generation sequencing data with FaQCs. BMC Bioinform..

[55] Li D., Liu C.M., Luo R., Sadakane K., Lam T.W. (2014). MEGAHIT: An ultra-fast single-node solution for large and complex metagenomics assembly via succinct de Bruijn graph. Bioinformatics.

[56] Li D., Luo R., Liu C.M., Leung C.M., Ting H.F., Sadakane K., Yamashita H., Lam T.W. (2016). MEGAHIT v1.0: A fast and scalable metagenome assembler driven by advanced methodologies and community practices. Methods.

[57] Li H. (2013). Aligning sequence reads, clone sequences and assembly contigs with BWA-MEM. arXiv.

[58] Li H., Handsaker B., Wysoker A., Fennell T., Ruan J., Homer N., Marth G., Abecasis G., Durbin R. (2009). The Sequence Alignment/Map format and SAMtools. Bioinformatics.

[59] Altschul S.F., Gish W., Miller W., Myers E.W., Lipman D.J. (1990). Basic local alignment search tool. J. Mol. Biol..

[60] Camacho C., Coulouris G., Avagyan V., Ma N., Papadopoulos J., Bealer K., Madden T.L. (2009). BLAST+: Architecture and applications. BMC Bioinform..

[61] Index of/pub/taxonomy/new_taxdump. ftp.ncbi.nlm.nih.gov/pub/taxonomy/new_taxdump.

[62] International Committee on Taxonomy of Viruses (ICTV). https://talk.ictvonline.org/.

[63] ViralZone. https://viralzone.expasy.org/5576.

[64] Research, Laboratory of Chemical Life Science, Kyoto University Bioinfomatics Center, I. for C. Virus-Host DB. https://www.genome.jp/virushostdb/.

[65] Nayfach S., Camargo A.P., Schulz F., Eloe-Fadrosh E., Roux S., Kyrpides N.C. (2020). CheckV assesses the quality and completeness of metagenome-assembled viral genomes. Nat. Biotechnol..

[66] Oksanen J., Kindt R., Legendre P., O’Hara B., Simpson G.L., Solymos P.M., Stevens M.H.H., Wagner H. (2008). The vegan package. Community Ecol. Packag..

[67] Hill M.O. (1973). Diversity and evenness: An unifying notation and its consequences. Ecology.

[68] Rényi A. (1961). On measures of entropy and information. Proceedings of the Fourth Berkeley Symposium on Mathematical Statistics and Probability.

[69] Margalef R. (1956). Información y diversidad específica en las comunidades de organismos. Investigación Pesquera.

[70] Magurran A.E., Henderson P.A. (2003). Explaining the excess of rare species in natural species abundance distributions. Nature.

[71] Tothmeresz B. (1995). Comparison of different methods for diversity ordering. J. Veg. Sci..

[72] Kumar S., Stecher G., Li M., Knyaz C., Tamura K. (2018). MEGA X: Molecular evolutionary genetics analysis across computing platforms. Mol. Biol. Evol..

[73] Darriba D., Taboada G.L., Doallo R., Posada D. (2012). jModelTest 2: More models, new heuristics and parallel computing. Nat. Methods.

[74] Darriba D., Taboada G.L., Doallo R., Posada D. (2011). ProtTest 3: Fast selection of best-fit models of protein evolution. Bioinformatics.

[75] Ronquist F., Huelsenbeck J.P. (2003). MrBayes 3: Bayesian phylogenetic inference under mixed models. Bioinformatics.

[76] Ronquist F., Teslenko M., van der Mark P., Ayres D.L., Darling A., Höhna S., Larget B., Liu L., Suchard M.A., Huelsenbeck J.P. (2012). MrBayes 3.2: Efficient bayesian phylogenetic inference and model choice across a large model space. Syst. Biol..

[77] Suchard M.A., Lemey P., Baele G., Ayres D.L., Drummond A.J., Rambaut A. (2018). Bayesian phylogenetic and phylodynamic data integration using BEAST 1.10. Virus Evol..

[78] Moens U., Calvignac-Spencer S., Lauber C., Ramqvist T., Feltkamp M.C.W., Daugherty M.D., Verschoor E.J., Ehlers B. (2017). ICTV virus taxonomy profile: Polyomaviridae. J. Gen. Virol..

[79] Smith D.B., Becher P., Bukh J., Gould E.A., Meyers G., Monath T., Muerhoff A.S., Pletnev A., Rico-Hesse R., Stapleton J.T. (2016). Proposed update to the taxonomy of the genera Hepacivirus and Pegivirus within the Flaviviridae family. J. Gen. Virol..

[80] Abudurexiti A., Adkins S., Alioto D., Alkhovsky S.V., Avšič-Županc T., Ballinger M.J., Bente D.A., Beer M., Bergeron É., Blair C.D. (2019). Taxonomy of the order Bunyavirales: Update 2019. Arch. Virol..

[81] Marklewitz M., Dutari L.C., Paraskevopoulou S., Page R.A., Loaiza J.R., Junglen S. (2019). Diverse novel phleboviruses in sandflies from the Panama Canal area, Central Panama. J. Gen. Virol..

[82] Nunes-Neto J.P., De Souza W.M., Acrani G.O., Romeiro M.F., Fumagalli M., Vieira L.C., De Almeida Medeiros D.B., Lima J.A., De Lima C.P.S., Cardoso J.F. (2017). Characterization of the bujaru, frijoles and tapara antigenic complexes into the sandfly fever group and two unclassified phleboviruses from Brazil. J. Gen. Virol..

[83] PhyloPic. http://phylopic.org.

[84] Wu Z., Han Y., Liu B., Li H., Zhu G., Latinne A., Dong J., Sun L., Du J., Zhou S. (2020). Decoding the RNA viromes of rodent lungs provides new visions into the origin and evolution pattern of rodent-borne diseases in Mainland Southeast Asia. Microbiome.

[85] Firth C., Bhat M., Firth M.A., Williams S.H., Frye M.J., Simmonds P., Conte J.M., Ng J., Garcia J., Bhuva N.P. (2014). Detection of zoonotic pathogens and characterization of novel viruses carried by commensal *Rattus norvegicus* in New York city. MBio.

[86] Phan T.G., Kapusinszky B., Wang C., Rose R.K., Lipton H.L., Delwart E.L. (2011). The fecal viral flora of wild rodents. PLoS Pathog..

[87] Nieto-Rabiela F., Suzán G., Wiratsudakul A., Rico-Chávez O. (2018). Viral metacommunities associated to bats and rodents at different spatial scales. Community Ecol..

[88] Campbell S.J., Ashley W., Gil-Fernandez M., Newsome T.M., Di Giallonardo F., Ortiz-Baez A.S., Mahar J.E., Towerton A.L., Gillings M., Holmes E.C. (2020). Red fox viromes in urban and rural landscapes. Virus Evol..

[89] Geoghegan J.L., Giallonardo F.D., Wille M., Ortiz-Baez A.S., Costa V.A., Ghaly T., Mifsud J.C.O., Turnbull O.M.H., Bellwood D.R., Williamson J.E. (2020). Host evolutionary history and ecology shape virome composition in fishes. bioRxiv.

[90] Sachsenröder J., Braun A., Machnowska P., Ng T.F.F., Deng X., Guenther S., Bernstein S., Ulrich R.G., Delwart E., Johne R. (2014). Metagenomic identification of novel enteric viruses in urban wild rats and genome characterization of a group A rotavirus. J. Gen. Virol..

[91] Sogin M.L., Morrison H.G., Huber J.A., Welch D.B.M.M., Huse S.M., Neal P.R., Arrieta J.M., Herndl G.J. (2006). Microbial diversity in the deep sea and the underexplored “rare biosphere. Proc. Natl. Acad. Sci. USA.

[92] Guillotin M. (1982). Rythmes d’activité et régimes alimentaires de *Proechimys cuvieri* et d’*Oryzomys capito velutinus* (Rodentia) en forêt guyanaise. Rev. Ecol. (Terre Vie).

[93] Tier A., de Carvalho W.D., Rostain S., Catzeflis F., Claessens O., Dewynter M., McKey D., Mustin K., Palisse M., de Thoisy B. (2020). The amazonian savannas of French Guiana: Cultural and social importance, biodiversity, and conservation challenges. Trop. Conserv. Sci..

[94] Solbrig O.T. (1996). The diversity of the savanna ecosystem. Ecol. Stud..

[95] Gubler D.J. (2002). The global emergence/resurgence of arboviral diseases as public health problems. Arch. Med. Res..

[96] Machalaba C., Karesh W.B. (2017). Emerging infectious disease risk: Shared drivers with environmental change. OIE Rev. Sci. Technol..

[97] Tilman D., May R.M., Lehman C.L., Nowack M.A. (1994). Habitat destruction and the extinction debt revisited. Nature.

[98] Ostfeld R.S., Keesing F. (2000). Biodiversity and disease risk: The case of Lyme disease. Conserv. Biol..

[99] Schmid J., Rasche A., Eibner G., Jeworowski L., Page R.A., Corman V.M., Drosten C., Sommer S. (2018). Ecological drivers of Hepacivirus infection in a neotropical rodent inhabiting landscapes with various degrees of human environmental change. Oecologia.

[100] Suzán G., Marcé E., Giermakowski J.T., Mills J.N., Ceballos G., Ostfeld R.S., Armién B., Pascale J.M., Yates T.L. (2009). Experimental evidence for reduced rodent diversity causing increased hantavirus prevalence. PLoS ONE.

[101] Maia F.G.M., de Souza W.M., Sabino-Santos G., Fumagalli M.J., Modha S., Murcia P.R., Figueiredo L.T.M. (2018). A novel polyomavirus in sigmodontine rodents from São Paulo State, Brazil. Arch. Virol..

[102] da Silva M.S., Cibulski S.P., Alves C.D.B.T., Weber M.N., Budaszewski R.F., Silveira S., Mósena A.C.S., Mayer F.Q., Goltz L.V., Campos R. (2018). New polyomavirus species identified in nutria, *Myocastor coypus* polyomavirus 1. Arch. Virol..

[103] Moens U., Krumbholz A., Ehlers B., Zell R., Johne R., Calvignac-Spencer S., Lauber C. (2017). Biology, evolution, and medical importance of polyomaviruses: An update. Infect. Genet. Evol..

[104] Calvignac-Spencer S., Feltkamp M.C.W., Daugherty M.D., Moens U., Ramqvist T., Johne R., Ehlers B. (2016). A taxonomy update for the family *Polyomaviridae*. Arch. Virol..

[105] Ehlers B., Anoh A.E., Salem N.B., Broll S., Couacy-Hymann E., Fischer D., Gedvilaite A., Ingenhütt N., Liebmann S., Martin M. (2019). Novel polyomaviruses in mammals from multiple orders and reassessment of polyomavirus evolution and taxonomy. Viruses.

[106] Tan Z., Gonzalez G., Sheng J., Wu J., Zhang F., Xu L., Zhang P., Zhu A., Qu Y., Tu C. (2020). Extensive genetic diversity of Polyomaviruses in sympatric bat communities: Host switching versus coevolution. J. Virol..

[107] Carr M., Gonzalez G., Sasaki M., Dool S.E., Ito K., Ishii A., Hang’ombe B.M., Mweene A.S., Teeling E.C., Hall W.W. (2017). Identification of the same polyomavirus species in different African horseshoe bat species is indicative of short-range host-switching events. J. Gen. Virol..

[108] Rasche A., Sander A.L., Corman V.M., Drexler J.F. (2019). Evolutionary biology of human hepatitis viruses. J. Hepatol..

[109] Shi M., Lin X.D., Tian J.H., Chen L.J., Chen X., Li C.X., Qin X.C., Li J., Cao J.P., Eden J.S. (2016). Redefining the invertebrate RNA virosphere. Nature.

[110] Li L.L., Liu M.M., Shen S., Zhang Y.J., Xu Y.L., Deng H.Y., Deng F., Duan Z.J. (2019). Detection and characterization of a novel hepacivirus in long-tailed ground squirrels (*Spermophilus undulatus*) in China. Arch. Virol..

[111] Kapoor A., Simmonds P., Scheel T.K.H., Hjelle B., Cullen J.M., Burbelo P.D., Chauhan L.V., Duraisamy R., Sanchez Leon M., Jain K. (2013). Identification of rodent homologs of hepatitis C virus and pegiviruses. MBio.

[112] Drexler J.F., Corman V.M., Müller M.A., Lukashev A.N., Gmyl A., Coutard B., Adam A., Ritz D., Leijten L.M., van Riel D. (2013). Evidence for Novel Hepaciviruses in Rodents. PLoS Pathog..

[113] de Souza W., Fumagalli M., Sabino-Santos G., Motta Maia F., Modha S., Teixeira Nunes M., Murcia P., Moraes Figueiredo L. (2019). A novel Hepacivirus in wild rodents from South America. Viruses.

[114] Scheel T.K.H., Simmonds P., Kapoor A. (2015). Surveying the global virome: Identification and characterization of HCV-related animal hepaciviruses. Antivir. Res..

[115] Pfaender S., Brown R.J.P., Pietschmann T., Steinmann E. (2014). Natural reservoirs for homologs of hepatitis C virus. Emerg. Microbes Infect..

[116] Pybus O.G., Gray R.R. (2013). The virus whose family expanded. Nature.

[117] Weaver S.C., Winegar R., Manger I.D., Forrester N.L. (2012). Alphaviruses: Population genetics and determinants of emergence. Antivir. Res..

[118] Aguilar P.V., Greene I.P., Coffey L.L., Medina G., Moncayo A.C., Anishchenko M., Ludwig G.V., Turell M.J., O’Guinn M.L., Lee J. (2004). Endemic Venezuelan Equine Encephalitis in northern Peru. Emerg. Infect. Dis..

[119] Rico-Hesse R., Weaver S.C., De Siger J., Medina G., Salas R.A. (1995). Emergence of a new epidemic/epizootic Venezuelan Equine Encephalitis virus in South America. Proc. Natl. Acad. Sci. USA.

[120] Rivas F., Diaz L.A., Cardenas V.M., Daza E., Bruzon L., Alcala A., De La Hoz O., Caceres F.M., Aristizabal G., Martinez J.W. (1997). Epidemic Venezuelan Equine Encephalitis in La Guajira, Colombia, 1995. J. Infect. Dis..

[121] Taylor K.G., Paessler S. (2013). Pathogenesis of Venezuelan Equine Encephalitis. Vet. Microbiol..

[122] Weaver S.C., Ferro C., Barrera R., Boshell J., Navarro J.C. (2004). Venezuelan Equine Encephalitis. Annu. Rev. Entomol..

[123] Phillpotts R.J., O’Brien L., Appleton R.E., Carr S., Bennett A. (2005). Intranasal immunisation with defective adenovirus serotype 5 expressing the Venezuelan Equine Encephalitis virus E2 glycoprotein protects against airborne challenge with virulent virus. Vaccine.

[124] Hommel D., Heraud J.M., Hulin A., Talarmin A. (2000). Association of Tonate virus (subtype IIIB of the Venezuelan Equine Encephalitis complex) with encephalitis in a human. Clin. Infect. Dis..

[125] Monath T.P., Lazuick J.S., Cropp C.B., Rush W.A., Calisher C.H., Kinney R.M., Trent D.W., Kemp G.E., Bowen G.S., Francy D.B. (1980). Recovery of Tonate virus (“bijou bridge” strain), a member of the Venezuelan Equine Encephalomyelitis virus complex, from Cliff Swallow nest bugs (*Oeciacus vicarius*) and nestling birds in North America. Am. J. Trop. Med. Hyg..

[126] Carrara A.S., Gonzales M., Ferro C., Tamayo M., Aronson J., Paessler S., Anishchenko M., Boshell J., Weaver S.C. (2005). Venezuelan Equine Encephalitis virus infection of spiny rats. Emerg. Infect. Dis..

[127] Deardorff E.R., Forrester N.L., Travassos Da Rosa A.P., Estrada-Franco J.G., Navarro-Lopez R., Tesh R.B., Weaver S.C. (2009). Experimental infection of potential reservoir hosts with Venezuelan Equine Encephalitis virus, Mexico. Emerg. Infect. Dis..

[128] Mutricy R., Djossou F., Matheus S., Lorenzi-Martinez E., De Laval F., Demar M., Nacher M., Rousset D., Epelboin L. (2020). Discriminating Tonate Virus from Dengue virus infection: A matched case–control study in French Guiana, 2003–2016. Am. J. Trop. Med. Hyg..

[129] Aguilar P.V., Estrada-Franco J.G., Navarro-Lopez R., Ferro C., Haddow A.D., Weaver S.C. (2011). Endemic Venezuelan Equine Encephalitis in the Americas: Hidden under the dengue umbrella. Future Virol..

[130] De Carvalho M.S., De Lara Pinto A.Z., Pinheiro A., Rodrigues J.S.V., Melo F.L., Da Silva L.A., Ribeiro B.M., Dezengrini-Slhessarenko R. (2018). Viola Phlebovirus is a novel Phlebotomus fever serogroup member identified in *Lutzomyia* (*Lutzomyia*) *longipalpis* from Brazilian Pantanal. Parasites Vectors.

[131] Palacios G., Wiley M.R., Travassos da Rosa A.P.A., Guzman H., Quiroz E., Savji N., Carrera J.P., Bussetti A.V., Ladner J.T., Ian Lipkin W. (2015). Characterization of the Punta Toro species complex (genus *Phlebovirus*, family *Bunyaviridae*). J. Gen. Virol..

[132] Gundacker N.D., Carrera J.P., Castillo M., Díaz Y., Valenzuela J., Tamhane A., Moreno B., Pascale J.M., Tesh R.B., López-Vergès S. (2017). Clinical manifestations of Punta Toro virus species complex infections, Panama, 2009. Emerg. Infect. Dis..

[133] Lorenz C., de Oliveira Lage M., Chiaravalloti-Neto F. (2021). Deforestation hotspots, climate crisis, and the perfect scenario for the next epidemic: The Amazon time bomb. Sci. Total Environ..

