# Supplementary material for: The Influence of Habitat on Viral Diversity in Neotropical Rodent Hosts
Source: Viruses. 2021 Aug 26;13(9):1690. doi: 10.3390/v13091690 (PMC8472065; doi:10.3390/v13091690)
Supplement: Supplementary file 1 [file viruses-13-01690-s001.zip › viruses-1346345 - table S1 from proof Katarina.pdf]

Table S.1. Number of viral read of each viral genus/subfamily according rodent species and habitats.

Species ->	*Proechimys guyannensis*		*Proechimys cuvieri*		*Hylaeamys megacephalus*		*Zygodontomys brevicauda*			Total number of reads
Habitats ->	Pristine forest	Disturbed forest	Pristine forest	Disturbed forest	Pristine forest	Disturbed forest	Savannah	Disturbed forest	Peri-Urban areas	
Genus/subfamily										
Cafeteriavirus	5876	0	0	0	0	0	0	0	0	5876
Mivirus	0	0	0	1959	0	60	0	0	0	2019
Iflavirus	0	0	0	0	0	30	0	0	0	30
unclassifiedNudiviridae	120	0	0	0	0	0	0	0	0	120
unclassifiedPicornaviridae	0	0	0	0	0	15	0	0	0	15
Sopolycivirus	0	0	0	0	0	66	20	85	0	171
unclassifiedPolycipiviridae	0	0	0	0	0	352	352	263	0	967
Potexvirus	42	0	0	0	0	0	0	0	0	42
Caulimovirus	0	0	0	28	0	0	0	0	0	28
Polerovirus	0	0	0	0	38	0	0	0	0	38
Betapartitivirus	0	0	17	0	0	0	0	0	0	17
unclassifiedPartitiviridae	18	0	0	0	0	0	0	0	0	18
unclassifiedPhycodnaviridae	11	0	0	0	0	15	0	0	0	26
Procedovirinae	0	0	0	0	4747	0	0	0	32	4779
Mastadenovirus	0	22	0	0	0	0	0	0	0	22
Alphatorquevirus	0	0	0	0	0	0	0	44	0	44
Deltatorquevirus	82	137837	69083	3270	20	0	0	0	0	210292
Iotatorquevirus	0	82932	0	367	0	0	0	0	0	83299
unclassifiedAnelloviridae	66972	2948092	3400728	293092	217366	8018389	8386910	12760014	16741873	52833436
Mammarenavirus	0	0	0	0	37	0	0	0	0	37
unclassifiedArenaviridae	0	0	0	0	355	0	0	0	0	355
Equarterivirinae	0	272	0	0	0	0	0	0	0	272
Simarterivirinae	0	24	0	0	0	0	0	0	0	24
unclassifiedAstroviridae	19	37	22	27	218	0	35	44	0	402

Circovirus	15497	172	30	0	0	384	74671	57244	445	148443
Cyclovirus	0	0	0	0	0	574	969	836	1069	3448
unclassifiedCircoviridae	3947	0	0	0	0	2319	1151445	544892	7905	1710508
Hepacivirus	403185	5999	3285699	1004998	583	390671	801	860	1663	5094459
Pegivirus	47385	199	169	0	0	62	0	0	0	47815
unclassifiedFlaviviridae	2413	0	187	0	0	27	0	0	0	2627
Rubivirus	0	0	184	0	0	0	0	0	0	184
Densovirinae	0	0	0	0	0	29	0	0	0	29
Parvovirinae	7995	0	28	461	96	24	35	68	60	8767
unclassifiedParvoviridae	122	0	0	0	21	0	0	0	0	143
Alphapolyomavirus	0	114	0	0	0	0	0	0	0	114
Betapolyomavirus	0	0	0	0	0	6344	0	0	0	6344
Deltapolyomavirus	0	0	0	0	0	520	0	0	0	520
unclassifiedPolyomaviridae	0	0	0	0	0	83696	14	58	0	83768
Gammaretrovirus	0	0	37	0	200	0	0	0	0	237
unclassifiedRetroviridae	33	0	0	0	20	0	0	0	0	53
Phlebovirus	0	0	0	373	0	0	0	0	0	373
unclassifiedRhabdoviridae	0	0	0	127	0	0	0	0	0	127
Alphavirus	0	0	0	1695	0	0	0	0	0	1695
Gemycircularvirus	0	21144	1986	0	0	0	0	0	129	23259
Gemyduguivirus	0	2981	0	0	0	0	0	0	0	2981
Gemygorvirus	0	0	0	0	0	30	18	0	129	177
Gemykibivirus	1722	3426	11706	34	8024	80	1712	0	14413	41117
Gemykolovirus	0	45	0	0	0	0	0	0	0	45
Gemykrogvirus	0	0	0	0	0	54	0	0	0	54
unclassifiedGenomoviridae	0	15922	180	0	0	0	0	0	0	16102
**Total number of reads**	**555439**	**3219218**	**6770056**	**1306431**	**231725**	**8503741**	**9616982**	**13364408**	**16767718**	**60335718**
**Richness**	**17**	**16**	**14**	**12**	**13**	**21**	**12**	**11**	**10**	**50**
