# Supplementary material for: The Influence of Habitat on Viral Diversity in Neotropical Rodent Hosts
Source: Viruses. 2021 Aug 26;13(9):1690. doi: 10.3390/v13091690 (PMC8472065; doi:10.3390/v13091690)
Supplement: Supplementary file 1 [file viruses-13-01690-s001.zip › viruses-1346345 - table S2 from proof Katarina.pdf]

Table S.2. Sampling variables and viral richness for the nine species-habitat at organ level. The number of rodent individuals per sample, the number of viral genera/subfamily detected, the sequencing plateform and organ types are given below.

P.guy.PF: *Proechimys guyannensis* from Pristine forest, **P.guy.DF:** *Proechimys guyannensis* from Disturbed forest, **P.cuv.PF:** *Proechimys cuvieri* from Pristine forest, **P.cuv.DF:** *Proechimys cuvieri* from Disturbed forest, **H.meg.PF:** *Hylaeamys megacephalus* from Pristine forest, **H.meg.DF:** *Hylaeamys megacephalus* from Pristine forest, **Z.bre.SV:** *Zygodontomys brevicauda* from savannah, **Z.bre.DF:** *Zygodontomys brevicauda* from Disturbed forest, **Z.bre.PU:** *Zygodontomys brevicauda* from Peri-Urban areas.

Organ	# individual rodents	#viral genra	Sequencing Plateform	Organ Type
**k_Pguy_PF**	5	8	HiSeq2500	kidney
**r_Pguy_PF**	8	4	HiSeq2500	spleen
**s_Pguy_PF**	12	13	HiSeq2500	serum
**k_Pguy_DF**	24	8	HiSeq2500	kidney
**r_Pguy_DF**	27	7	HiSeq2500	spleen
**s_Pguy_DF**	27	9	HiSeq2500	serum
**k_Pcuv_PF**	9	8	HiSeq2500	kidney
**r_Pcuv_PF**	24	3	HiSeq2500	spleen
**s_Pcuv_PF**	31	10	HiSeq2500	serum
**k_Pcuv_DF**	18	5	HiSeq2500	kidney
**r_Pcuv_DF**	18	11	HiSeq2500	spleen
**s_Pcuv_DF**	15	6	HiSeq2500	serum
**k_Hmeg_PF**	2	5	HiSeq2500	kidney
**r_Hmeg_PF**	4	7	HiSeq2500	spleen
**s_Hmeg_PF**	3	6	HiSeq2500	serum
**k_Hmeg_DF**	4	12	MiSeq	kidney
**r_Hmeg_DF**	4	10	MiSeq	spleen
**s_Hmeg_DF**	4	6	HiSeq2500	serum

**k_Zbre_SV**	6	9	MiSeq	kidney
**r_Zbre_SV**	6	6	MiSeq	spleen
**s_Zbre_SV**	8	4	HiSeq2500	serum
**k_Zbre_DF**	7	10	MiSeq	kidney
**r_Zbre_DF**	7	7	MiSeq	spleen
**s_Zbre_DF**	10	2	HiSeq2500	serum
**k_Zbre_PU**	20	5	HiSeq2500	kidney
**r_Zbre_PU**	20	6	HiSeq2500	spleen
**s_Zbre_PU**	19	8	HiSeq2500	serum
