# Supplementary material for: The Influence of Habitat on Viral Diversity in Neotropical Rodent Hosts
Source: Viruses. 2021 Aug 26;13(9):1690. doi: 10.3390/v13091690 (PMC8472065; doi:10.3390/v13091690)
Supplement: Supplementary file 1 [file viruses-13-01690-s001.zip › viruses-1346345 - table S3 from proof Katarina.pdf]

Table S.4. Assembly statistics of each sample by organ species and habitat. The minimal length of contigs for assembly is 200 bp. Table shows number of contigs, the total length obtained after assembly, the maximum contigs length, the average contigs length and the N50 (length of the contig at 50% of the total length). The total, mean and median of all assembly statistics are indicated at the end of the table.The minimum and maximum values of each statistics are given in bold.

k : Kidney; r = spleen; s = serum; P.guy.PF: *Proechimys guyannensis* from Pristine forest, **P.guy.DF:** *Proechimys guyannensis* from Disturbed forest, **P.cuv.PF:** *Proechimys cuvieri* from Pristine forest, **P.cuv.DF:** *Proechimys cuvieri* from Disturbed forest, **Oauy_DF** : Oecomys auyantepui from Disturbed forest, **Obic_DF** : Oecomys bicolor from Disturbed forest, **H.meg.PF:** *Hylaeamys megacephalus* from Pristine forest, **H.meg.DF:** *Hylaeamys megacephalus* from Pristine forest, **Hyun_DF : Hylaemys yunganus from** Disturbed forest**, Z.bre.SV:** *Zygodontomys brevicauda* from savannah, **Z.bre.DF:** *Zygodontomys brevicauda* from Disturbed forest, **Z.bre.PU:** *Zygodontomys brevicauda* from Peri-Urban areas.

**Sample ID**	**contigs**	**Total length**	**Max contig length**	**Average contig length**	**N50**
**k_Pguy_PF**	323,113	124,796,901	13,364	386	376
**k_Pguy_DF**	143,955	55,645,824	23,457	387	378
**k_Pcuv_PF**	267,539	98,918,691	5,530	370	373
**k_Pcuv_DF**	88,325	37,294,420	25,889	422	404
**k_Oauy_DF**	225,793	83,516,641	12,193	370	375
**k_Obic_DF**	31,337	10,364,233	5,282	331	**312**
**k_Hmeg_PF**	285,067	100,529,472	**3,302**	353	346
**k_Hmeg_DF**	53,664	18,126,365	5,527	338	318
**k_Hyun_DF**	159,779	57,332,105	5,527	359	365
**k_Zbre_PU**	181,251	63,769,797	5,801	352	364
**k_Zbre_DF**	56,113	21,634,921	**430,386**	386	320
**k_Zbre_SV**	**25,215**	**8,550,895**	8,611	339	312
**r_Pguy_PF**	330,777	121,001,588	5,316	366	366
**r_Pguy_DF**	231,108	85,463,485	6,113	370	368
**r_Pcuv_PF**	124,874	61,603,770	61,880	493	445
**r_Pcuv_DF**	267,976	128,197,750	28,919	478	442
**r_Oauy_DF**	184,913	69,280,188	20,011	375	372
**r_Obic_DF**	67,976	22,436,584	4,581	**330**	314
**r_Hmeg_PF**	282,402	97,531,492	5,527	345	340
**r_Hmeg_DF**	53,117	18,019,878	5,527	339	317
**r_Hyun_DF**	159,882	56,455,917	11,052	353	356
**r_Zbre_PU**	176,663	61,235,823	7,529	347	358
**r_Zbre_DF**	59,922	20,212,433	6,526	337	317
**r_Zbre_SV**	79,251	26,444,484	5,527	334	315
**s_Pguy_PF**	56,461	22,848,566	26,375	405	402
**s_Pguy_DF**	54,835	20,650,944	35,452	377	379

**s_Pcuv_PF**	86,555	35,480,275	25,400	410	412
**s_Pcuv_DF**	**347,957**	**130,212,578**	26,110	374	368
**s_Oauy_DF**	71,707	28,278,856	6,029	394	416
**s_Obic_DF**	103,205	60,641,996	91,235	**588**	**533**
**s_Hmeg_PF**	75,533	32,244,906	50,954	427	411
**s_Hmeg_DF**	70,539	30,442,191	42,743	432	419
**s_Hyun_DF**	258,141	124,081,961	17,327	481	479
**s_Zbre_PU**	87,791	33,009,025	92,987	376	378
**s_Zbre_DF**	103,974	37,981,911	17,599	365	363
**s_Zbre_SV**	91,402	34,782,756	40,679	381	377
TOTAL	**5,268,112**	**2,039,019,622**	-	-	-
MEAN	**146,336**	**56,639,434**	**32,952**	**385**	**375**
MEDIAN	**103,590**	**46,813,868**	**12,779**	**372**	**370**
