# Supplementary material for: The Influence of Habitat on Viral Diversity in Neotropical Rodent Hosts
Source: Viruses. 2021 Aug 26;13(9):1690. doi: 10.3390/v13091690 (PMC8472065; doi:10.3390/v13091690)
Supplement: Supplementary file 1 [file viruses-13-01690-s001.zip › viruses-1346345 - table S4 from proof Katarina.pdf]

Table S.4. Number of viral contigs by rodent species/habitats and viral families.

Total number of contigs are given for each species habitat and across species habitats. Genome types are mentioned along the viral family names as follow ** = dsDNA, * = ssDNA, ++ = dsRNA, + = ssRNA (+), - = ssRNA (-), °° = DNA-retrotranscribing, ° = RNA-retrotranscribing. Left row represent host-type (Vertebrate, Invertebrate, Plants…).

Host	Rodent species	*Proechimys guyannensis*		*Proechimys cuvieri*		*Oecomys auyantepui*	*Oecomys bicolor*	*Hylaeamys yunganus*	*Hylaeamys megacephalus*		*Zygodontomys brevicauda*			
	Environments / Viral family	Pristine forest	Disturbed forest	Pristine forest	Disturbed forest	Disturbed forest	Disturbed forest	Disturbed forest	Pristine forest	Disturbed forest	Savannah	Disturbed forest	Peri-urban	Total
Amoeba	*Mimiviridae* **	18	0	0	0	0	0	0	0	0	0	0	0	18
Invertebrates	*Chuviridae* -	0	0	0	3	0	0	0	0	2	0	0	0	
	Iflaviridae +	0	0	0	0	0	0	0	0	1	0	0	0	
	Nudiviridae **	4	0	0	0	0	0	0	0	0	0	0	0	
	Picornaviridae +	0	0	0	0	0	0	0	0	1	0	0	0	
	Polycipiviridae +	0	0	0	0	0	7	0	0	12	9	10	0	
	**Total**	4	0	0	3	0	7	0	0	16	9	10	0	49
Plants	*Alphaflexiviridae* +	1	0	0	0	0	0	0	0	0	0	0	0	
	Caulimoviridae °°	0	0	0	1	0	0	0	0	0	0	0	0	
	Luteoviridae +	0	0	0	0	0	1	0	1	0	0	0	0	

	***Partitiviridae* ++**	1	0	1	0	0	0	0	0	0	0	0	0	
	Phycodnaviridae* *	1	0	0	0	0	1	0	0	0	0	0	0	
	***Tombusviridae* +**	0	0	0	0	1	76	1	19	0	0	0	1	
	**Total**	**3**	**0**	**1**	**1**	**1**	**78**	**1**	**20**	**0**	**0**	**0**	**1**	106
Vertebrates-strict	***Adenoviridae* ****	0	1	0	0	0	0	0	0	0	0	0	0	
	***Anelloviridae* ***	160	2906	1932	337	195	5379	4992	161	4174	6976	12671	19004	
	***Arenaviridae* -**	0	0	0	0	1	0	0	4	0	0	0	0	
	***Arteriviridae* +**	0	1	0	0	0	0	0	0	0	0	0	0	
	***Astroviridae* +**	1	2	4	1	2	1	0	2	0	1	2	0	
	***Circoviridae* ***	12	1	2	0	0	1095	0	0	22	442	118	9	
	***Flaviviridae* +**	1728	41	9830	3514	63	2	39	17	1365	21	20	46	
	***Matonaviridae* +**	0	0	2	0	0	0	0	0	0	0	0	0	
	***Parvoviridae* ***	59	0	1	3	0	0	1	3	2	1	2	2	
	***Polyomaviridae* ***	0	3	0	0	0	1	0	0	46	2	2	0	
	***Retroviridae* °**	1	0	2	0	2	0	3	3	0	0	0	0	
	**Total**	**1961**	**2955**	**11773**	**3855**	**263**	**6478**	**5035**	**190**	**5609**	**7443**	**12815**	**19061**	77438
	***Phenuiviridae* -**	0	0	0	4	0	0	0	0	0	0	0	0	
	**Rhabdoviridae -**	0	0	0	1	0	0	0	0	0	0	0	0	

	Togaviridae +	0	0	0	14	0	0	0	0	0	0	0	0	
	Total	0	0	0	19	0	0	0	0	0	0	0	0	19
Vert. Invert. Fungi	*Genomoviridae* *	2	79	7	1	10	0	1	8	4	5	0	20	137
	TOTAL	1988	3034	11781	3879	274	6563	5037	218	5629	7457	12825	19082	77767
